# Preconditioning With TGF‐β Inhibitors Enhances Therapeutic Efficacy of Endothelial Progenitor Cells for Wound Healing in Diabetic Mice

**DOI:** 10.1002/mco2.70364

**Published:** 2025-09-01

**Authors:** Dongsheng Su, Fuyi Cheng, Qingyuan Jiang, Yong Zhang, Fei Du, Cheng Pan, Yixin Ye, Lin Zhang, Pusong Zhao, Huilin Wang, Qi Xiong, Xiaolan Su, Hongxin Deng

**Affiliations:** ^1^ Department of Biotherapy Cancer Center and State Key Laboratory of Biotherapy West China Hospital Sichuan University Chengdu Sichuan People's Republic of China; ^2^ Department of Plastic Reconstructive and Aesthetic Surgery, West China Second University Hospital, Sichuan University/West China Women's and Children's Hospital Chengdu Sichuan People's Republic of China; ^3^ Department of Obstetrics Sichuan Provincial Hospital For Women and Children Chengdu Sichuan People's Republic of China

**Keywords:** angiogenesis, cell therapy, diabetic wound, endothelial progenitor cells, preconditioning, TGF‐β signaling

## Abstract

Diabetic wound (DW) represent a common complication of diabetes. Despite advances in regenerative repair utilizing endothelial progenitor cells (EPCs), challenges such as low survival and impaired angiogenic function of EPCs remain. Herein, we explored an effective method to induce injury‐induced protection for EPCs and improves their function. This was achieved through cell preconditioning under conditions of nutrient deprivation and high glucose (NDHG), combined with sb431542, a transforming growth factor beta (TGF‐β) signaling inhibitor. Specifically, after three generations of cell passage during preconditioning, umbilical cord‐derived endothelial cells (ECs) exhibited characteristics resembling those of EPCs, with over 80% of the cells expressed CD34, a typical marker of EPCs. Notably, these preconditioned EPC‐like cells (pEPCs) showed tolerance to pathological environment, as evidenced by robust cell viability, improved antioxidant capacity, and stable tube‐forming ability under NDHG condition. The protective effect of preconditioning in pEPCs is partly achieved by activating the PI3K/AKT pathway to upregulate the expression of Nrf2 and HIF‐1α. Importantly, pEPCs exhibited therapeutic potential in two diabetic mouse models‐limb ischemia and skin wounds by enhancing blood vessel formation and facilitating tissue repair. Overall, this preconditioning method induced the generation of functionally enhanced pEPCs, providing an alternative source of cells for treating DWs.

## Graphical abstract

In this study, we explore a novel and efficient preconditioning method that induced EPCs‐like cells from hUVECs. TGF‐β inhibitors–treated hUVECs demonstrated significantly enhanced cell activity and tube‐forming ability under nutrient‐deficient and high‐glucose‐containing diabetic wound simulation conditions. Furthermore, they exhibited the ability to repair limb and skin injuries in diabetic mice.

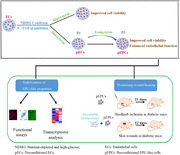

## 1 Introduction

The refractory diabetic wound (DW) is a serious complication of diabetes mellitus that leads to ulcerations and amputations of the lower extremities [1, 2]. During the healing of DW, impaired angiogenesis is partially responsible for the pathological process [3, 4, 5]. Despite the availability of standard revascularization treatments such as angioplasty, arterial bypass, and vascular growth factors, a high incidence of amputation remains [6]. Therefore, there is an urgent requirement to explore alternative therapeutic options for DW.

Stem cell‐based therapy has emerged as a potential treatment for DW. Clinical trials have reported that transplantation of CD34^+^ endothelial progenitor cells (EPCs), derived from bone marrow, can result in long‐term improvements of severe limb ischemia for patients who are not eligible for standard revascularization procedures [7, 8]. Nonetheless, the therapeutic efficacy of the transplanted EPCs is compromised by their poor survival in the high‐glucose and nutrient‐deficient pathological conditions of DW [9, 10]. Although introducing specific genes, such as CXCR7, Nampt, or microRNAs, into EPCs has shown promise in enhancing their function [9, 10, 11], the genetic manipulation of cells raises safety concerns and is not yet suitable for widespread therapeutic application.

Preconditioning has been shown to improve the resistance of transplanted cells to the challenging host microenvironment by subjecting them to conditions that mimic the harsh conditions of damaged tissues [12, 13]. Previous studies have reported various preconditioning schemes involving pharmacological and/or physical inducers to enhance the reparative potential of EPCs. For instance, pinocembrin preconditioning has been found to improve the therapeutic efficacy of EPCs in rats with monocrotaline‐induced pulmonary hypertension [14]. Ischemic preconditioning of EPCs has been shown to attenuate renal ischemia/reperfusion injury [15], and EPCs preconditioned with defined small molecules improve their therapy against ischemic diseases [16]. However, the optimal preconditioning scheme that can increase the proangiogenic responses of EPCs and maximize their therapeutic potential for DW remains to be elucidated.

The signaling pathway of transforming growth factor beta (TGF‐β) is critical for the function and plasticity regulation of ECs in health and diseases [17, 18]. Research has indicated that hyperglycemia increases TGF‐β signaling in ECs, leading to detrimental effects on their viability and cellular function by triggering endothelial‐to‐mesenchymal transition and oxidative stress [19]. Additionally, TGF‐β signaling plays a significant role in the development of hyperglycemia‐induced metabolic memory in ECs, resulting in long‐lasting damage [20]. These findings highlight the potential of inhibiting TGF‐β signaling to reverse the damage to EPCs under the pathological conditions of DW. However, many studies have demonstrated that TGF‐β inhibitors can facilitate the differentiation of stem cells into ECs and enhance the proliferation of induced ECs by inhibiting TGF‐β signaling [21]. The potential application of TGF‐β inhibitors in the preconditioning of EPCs is not yet fully understood, and the underlying mechanism remains uncertain.

In the present study, we aimed to investigate the optimal components of preconditioning conditions and develop an effective scheme for preconditioning to obtain EPC‐like cells capable of tolerating the high glucose and low nutrient stress in a DW microenvironment. We analyzed the biological characteristics of these preconditioning‐induced EPC‐like cells (pEPCs), explored the underlying mechanisms, and evaluated the therapeutic potential of pEPCs in promoting neovascularization and skin regenerative repair in diabetic mice.

## 2 Results

### 2.1 Preconditioning Cell With TGF‐β Inhibitor SB431542 Under NDHG Conditions Enhances Endothelial Viability

To find effective preconditioning methods that improve EC tolerance to harsh environments, we established the nutrient‐deficient and high glucose‐containing (NDHG) conditions that mimic the pathological condition observed in DW (Figure S1), and the viability of ECs decreased with the increasing concentration of d‐glucose. Particularly, when the glucose concentration reached 75 mM, an increased number of apoptotic cells were observed floating in the culture supernatant. These results suggest that NDHG preconditioning alone is ineffective for enhancing the survival of ECs.

**FIGURE 1:**
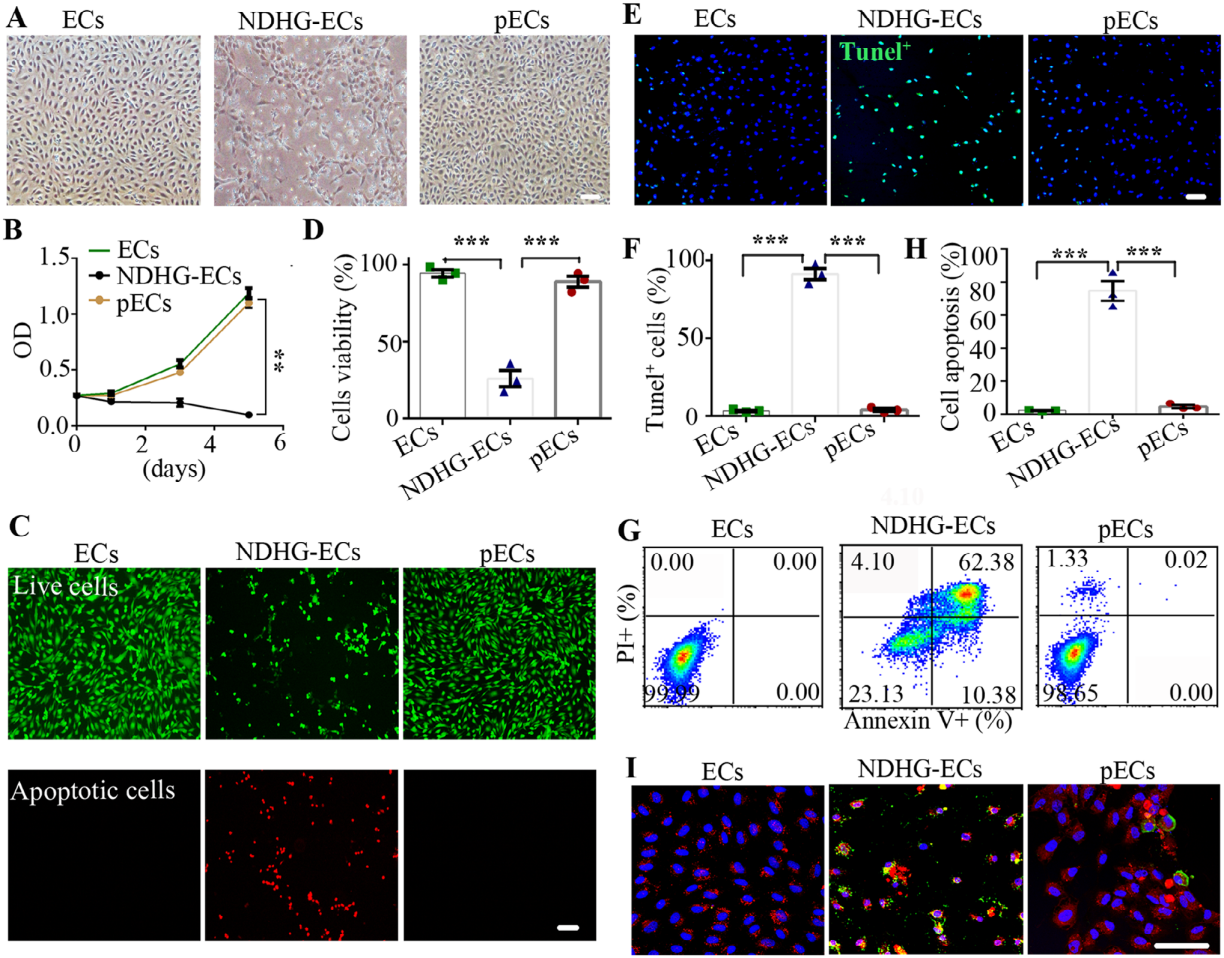
Sb‐NDHG preconditioning restores the viability of ECs under stressful conditions. (A) Representative brightfield images of cells cultured in different media for two days. (B) Cell viability was evaluated using the CCK‐8 assay. (C) Representative brightfield images of live/dead fluorescence staining. (D) Quantification of cell viability based on the live/dead staining results. (E) Cell apoptosis was tested by TUNEL staining. (F) Quantification of cell apoptosis based on the TUNEL staining results. (G) Cell apoptosis was assessed by flow cytometric analysis. (H) Quantification of cell apoptosis based on the flow cytometric analysis results. (I) Immunostaining of JC‐1 fluorescent probe (red) and Annexin V (green) in cells from different groups. Data are represented as mean ± SEM. **p < 0.01, ***p < 0.001 compared with the corresponding control. Scale bars represent 50 µm. ECs, hUVECs, NDHG‐ECs), NDHG‐preconditioned ECs; pECs, sb‐NDHG‐preconditioned ECs.

To improve the preconditioning conditions, we tested some small molecule compounds that have been reported to be beneficial for ECs [22, 23, 24]. Based on the results of cell viability (Figure S2), we identified the TGF‐β inhibitor sb431542 and its optimal concentration (Figure S3). We found that ECs cultured under NDHG conditions with sb431542 (hereafter referred to as sb‐NDHG) exhibited high cell viability and retained their cobblestone‐like morphology, comparable to parental ECs (Figure 1A,B). In our supplementation experiments, the activation of the TGF‐β signaling pathway in NDHG‐ECs supports the rationale for using sb431542 (Figure S4). This result was further confirmed by live/dead cell staining and the TUNEL staining (Figure 1C–F). Moreover, flow cytometry analysis revealed that the number of apoptotic cells in sb‐NDHG‐preconditioned ECs (pECs) decreased from nearly 70% to less than 5% compared to NDHG‐preconditioned ECs (NDHG‐ECs) (Figure 1G,H). Our further research demonstrates that the combination of NDHG conditions and sb431542 enhances EC survival, rather than sb431542 alone (Figure S5).

Changes of mitochondrial membrane potential (MMP) also serve as a crucial indicator of cell apoptosis. ECs stained with the MMP fluorescent probe (JC‐1) exhibited bright red fluorescence (Figure 1I). However, in NDHG‐ECs, the fluorescence intensity of JC‐1 decreased, while the green fluorescence (annexin V‐FITC+) increased. Conversely, the fluorescence intensity of JC‐1 in pECs was upregulated, indicating a recovery of cell viability. Collectively, these results indicated that preconditioning with sb‐NDHG could confer a survival advantage to ECs under stressful conditions.

While screening small molecular compounds, we also discovered that other TGF‐β inhibitors, including SB525334 and A83‐01, enhance EC viability under NDHG conditions (Figure S6). In our follow‐up study, we chose sb431542 to further investigate whether and how sb‐NDHG preconditioning affects ECs.

### 2.2 Sb‐NDHG Preconditioning Preserves Endothelial‐Specific Characteristics in pECs Under Stressful Conditions

EndMT is a complex process in which ECs reduce the expression of endothelial markers and undergo trans‐differentiation into myofibroblasts or mesenchymal cells involved in pathological fibrosis. This process can be robustly induced by hyperglycemia [25]. Although sb‐NDHG preconditioning has been shown to increase the viability of ECs, it remains unclear whether sb‐NDHG preconditioning can inhibit EndMT and preserve the original properties of ECs during long‐term culture.

**FIGURE 2:**
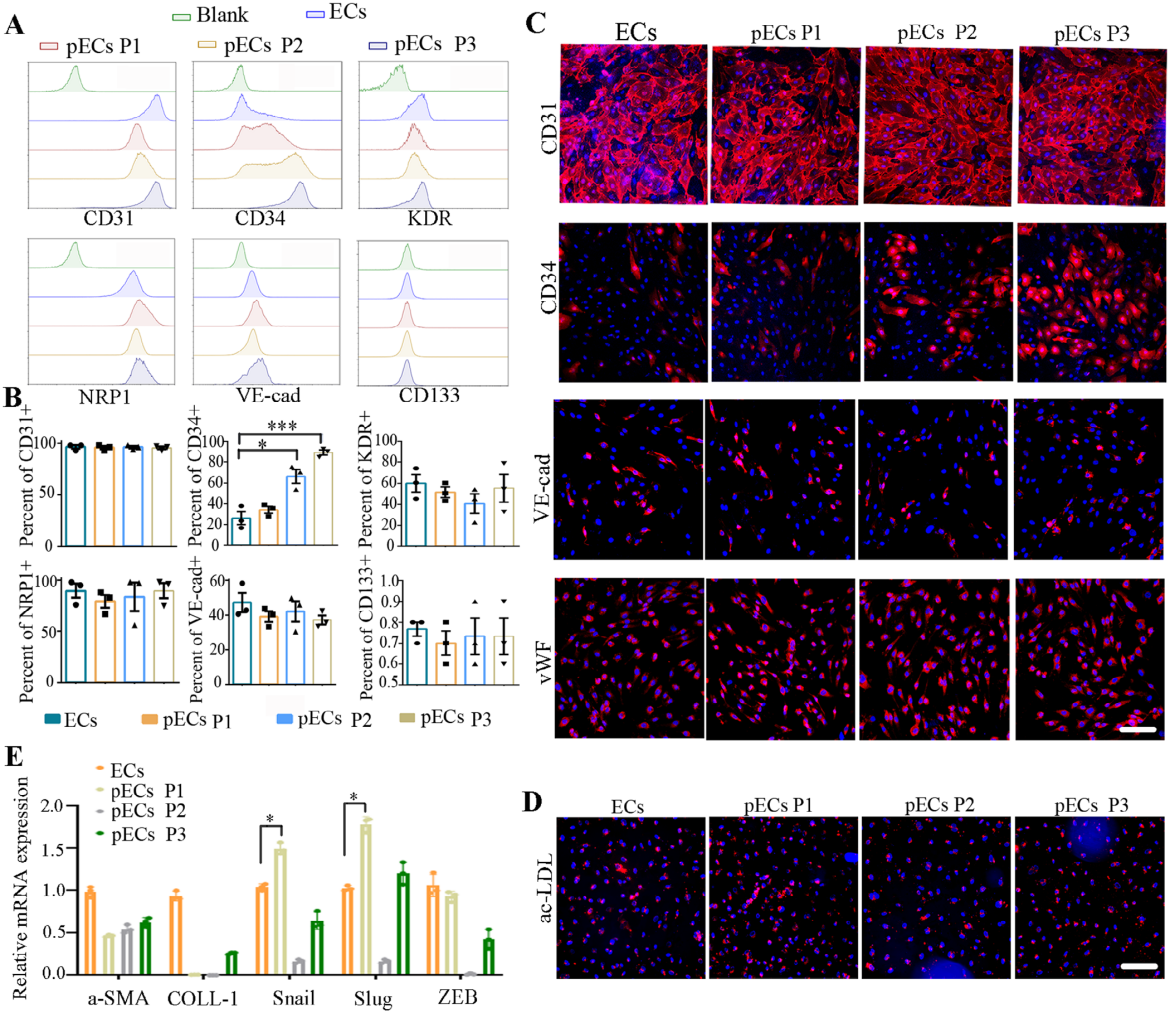
Sb‐NDHG preconditioning contributes to maintaining the endothelial‐specific characteristics of ECs. pECs were passaged three times and are defined as pECs P1, pECs P2, and pECs P3. (A) Endothelial surface markers were detected using flow‐cytometry analysis. (B) Quantification of endothelial surface markers based on the results of flow cytometry analysis. (C) Immunostaining of endothelial‐specific markers. (D) Dil‐acLDL uptake assay in the indicated cells. (E) EndMT signature genes were detected by qRT‐PCR. Data are represented as mean ± SEM. *p < 0.05, ***p < 0.001 compared with the corresponding control. Scale bars represent 50 µm.

To test this hypothesis, endothelial surface markers were detected using flow cytometry (Figure 2A). Compared with ECs, pECs expressed the same levels of CD31, VE‐cad, KDR, and NRP1, while displaying negative expression of CD133 (Figure 2B). Notably, the expression of CD34, a well‐known marker of EPCs, gradually increased from approximately 20% at passage 0 (P0) to nearly 80% at passage 3 (P3) in pECs. Immunofluorescent staining of pECs, from P1 to P3 revealed the presence of typical endothelial markers, such as CD31, VE‐cad, and vWF. Notably, CD34^+^ cells, initially scattered among pECs at passage 1 (P1), gradually increased to became the predominant cell type in pECs at passage 3 (P3) (Figure 2C).

Compared to ECs, pECs showed no difference in the uptake of Dil‐acetylated low‐density lipoprotein (ac‐LDL) (Figure 2D). In addition, EndMT signature genes, including *α‐SMA, COLL‐1, Snail, Slug*, and *ZEB*, were not significantly upregulated in pECs at P3 (Figure 2E). These results suggest that pECs can stably maintain endothelial‐specific properties under stressful conditions.

### 2.3 Sb‐NDHG Preconditioning Enhanced pECs Angiogenic Activities and Oxidative Stress Resistance Under Stressful Conditions

**FIGURE 3:**
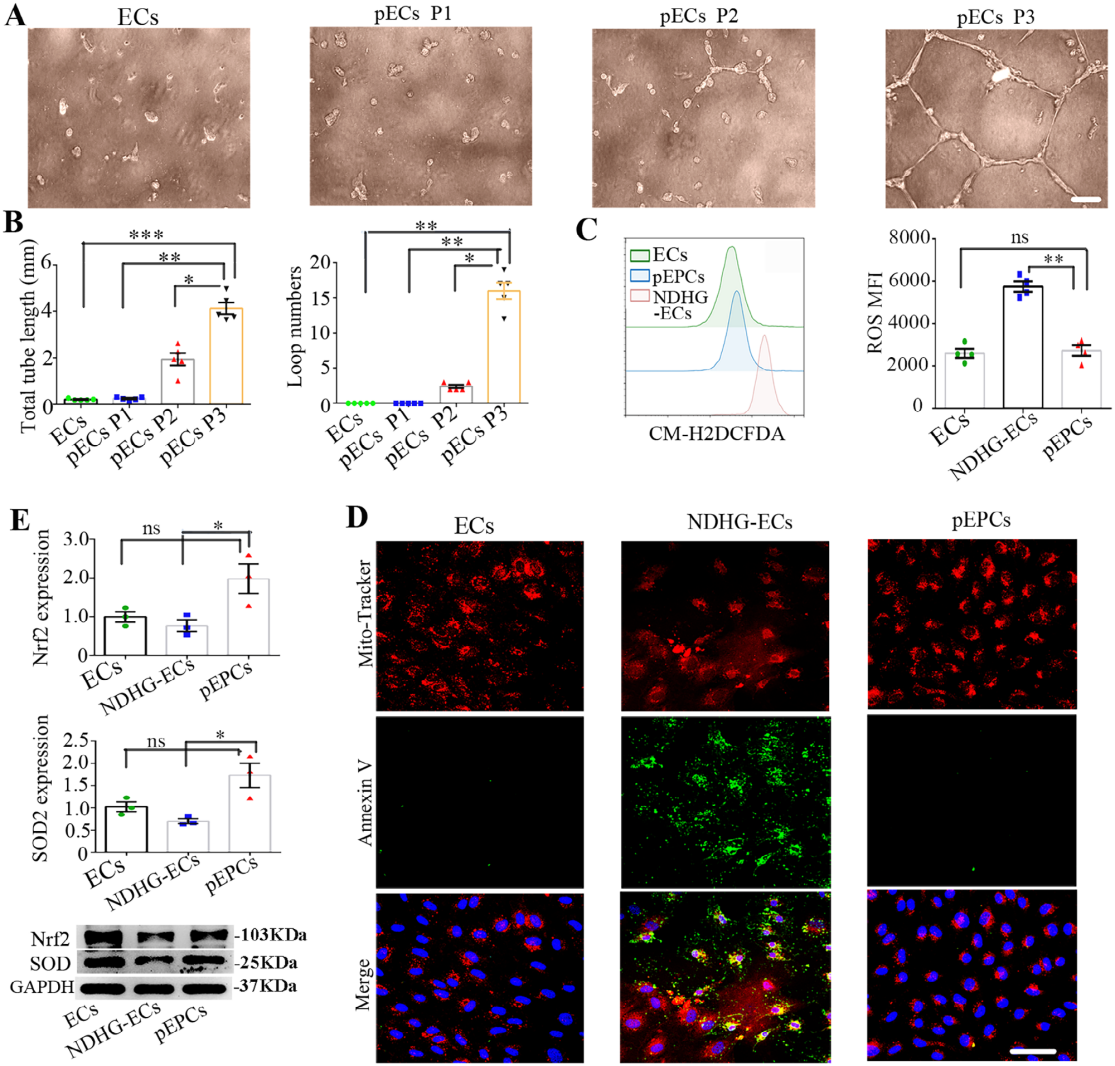
Long‐term Sb‐NDHG preconditioning facilitated the generation of pEPCs with enhanced endothelial function. (A) The tube formation ability of the indicated cells was evaluated under NDHG conditions. (B) Quantification of tube lengths and the number of loops was performed. (C) Flow cytometry analysis and quantification of ROS production in pEPCs were conducted. (D) Immunostaining of JC‐1 fluorescent probe (red) and annexin V (green) in pEPCs. (E) The mRNA levels (top) and the protein levels (bottom) of Nrf2 and SOD were tested. Data are represented as mean ± SEM. *p < 0.05, **p < 0.01, ***p < 0.001 compared with the corresponding control. Scale bars represent 50 µm. pEPCs, preconditioned EPC‐like cells

Angiogenesis is a hallmark of endothelial function and is seriously impaired in DW [26]. To evaluate the ability of angiogenesis, we conducted a Matrigel tube formation assay under NDHG conditions. Over a 12‐h observation period, ECs and pECs P1 failed to form any tubular structures, while only a few tube‐like structures were formed by pECs P2 (Figure 3A). Notably, pECs P3 exhibited a significant increase in both tube length and the number of loops in the tubular structures (Figure 3B). When cultured in endothelial growth medium (EGM), the tubular structures formed by pECs P3 were maintained for at least 44 h, which was longer than that of other cells (Figure S7). Based on their excellent tube‐formation ability and the high expression of CD34, pECs P3 could be considered as preconditioned EPC‐like cells (pEPCs) with enhanced endothelial function.

Hyperglycemia impairs EC function mainly through the accumulation of reactive oxygen species (ROS) [27]. The level of ROS in NDHG‐ECs was significantly higher than that in ECs (Figure 3C). The increased accumulation of ROS was linked to mitochondrial oxidative damage, which resulted in the loss of MMP in NDHG‐ECs (Figure 3D). Furthermore, the activity of nuclear factor erythroid 2 like 2 (Nrf2), a key transcription factor that regulates hundreds of genes involved in oxidative stress, was reduced [28] (Figure 3E). Consequently, the level of superoxide dismutase (SOD), a potent antioxidant transcribed from one of Nrf2's target genes, was also reduced, as analyzed by Quantitative Real‐time PCR (qRT‐PCR) and western blot (Figure 3E). Notably, the elevated ROS levels were significantly reduced in pEPCs, accompanied by the restoration of MMP as well as an increase in the expression levels of Nrf2 and SOD‐2 at both the mRNA and protein levels (Figure 3C–E). These findings demonstrate that sb‐NDHG preconditioning promotes the generation of pEPCs and enhances their endothelial function.

Two essential factors for cell‐based therapy are the angiogenic potential and large‐scale production of cell products. Our results indicate that pEPCs can expand in vitro for over five generations while maintaining stable biological properties (Figure S8). The pEPCs maintained their secretion of pro‐angiogenic cytokines at levels comparable to those of normal parental ECs (Figure S9). Furthermore, the processes of freezing and thawing did not have a detrimental effect on cell proliferation, the expression of cell surface markers, or tube formation ability (Figure S10). These findings strongly support pEPCs as a promising source of seed cells for regenerative repair therapies.

### 2.4 Protective Effects of sb‐NDHG Preconditioning on pEPCs via Partial Activation of the PI3K and Nrf2 Signaling Pathway

**FIGURE 4:**
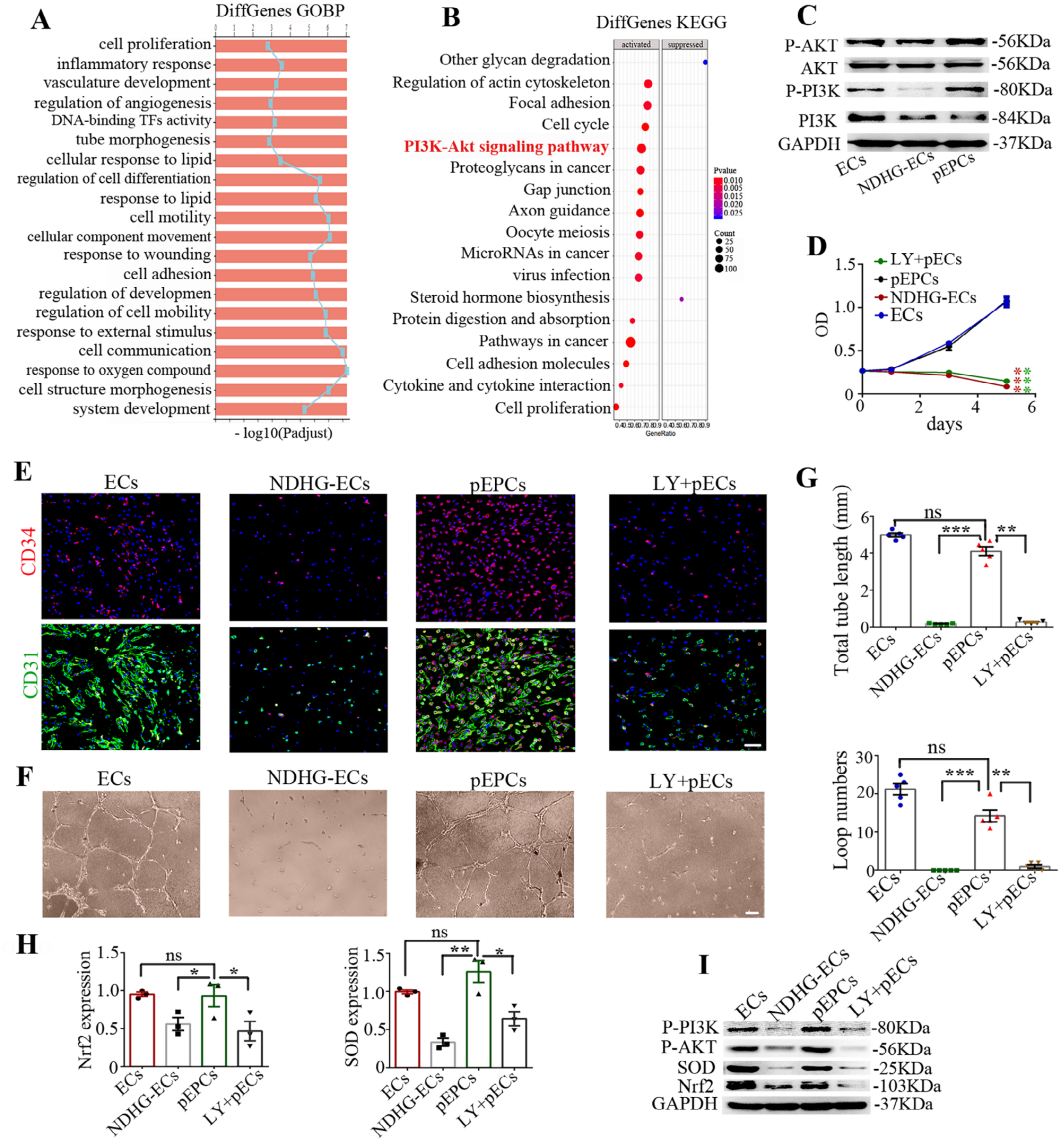
sb‐NDHG preconditioning enhances pEPC resilience through modulation of the PI3K‐Nrf2 pathway. (A) GO enrichment analysis of RNA‐seq data of DEGs between pEPCs and NDHG‐ECs. (B) KEGG pathway enrichment analysis of RNA‐seq data of DEGs between pEPCs and NDHG‐ECs. (C) Western blot was conducted to detect the expression of PI3K, P‐PI3K, AKT, and P‐AKT in pEPCs and NDHG‐ECs. (D) Cell viability was evaluated using the CCK‐8 assay. (E) Immunostaining of endothelia markers CD31 and CD34 in indicated cells. (F and G) Tube formation assay. Tube lengths and the loop number were measured 6 h after seeding under NDHG conditions. (H) mRNA levels of *Nrf2* and *SOD* were tested by qRT‐PCR. (I) Protein levels of Nrf2, SOD, P‐PI3K, and P‐AKT were determined by western blot analysis. Data are represented as mean ± SEM. *p < 0.05, **p < 0.01, ***p < 0.001 compared with the corresponding control. Scale bars represent 50 µm.

To gain insight into the molecular mechanism underlying the protective effects of sb‐NDHG preconditioning for pEPCs, we performed whole‐genome transcriptome analysis on ECs, NDHG‐ECs, and pEPCs. We identified the differentially expressed genes (DEGs) as those with *p* < 0.05 and a fold change > 2. Global gene expression analysis revealed that pEPCs clustered more closely with ECs than with NDHG‐ECs, as illustrated by hierarchical clustering (Figure S11A). To investigate the altered genes in cells, DEGs between pEPCs and NDHG‐ECs were subjected to Gene Ontology (GO) analysis. The DEGs were primarily associated with cell proliferation, vasculature development, regulation of angiogenesis, and tube morphogenesis (Figure 4A). To identify the signaling pathways associated with these effects, we conducted a Kyoto Encyclopedia of Genes and Genomes (KEGG) pathway enrichment analysis on the identified DEGs. The PI3K‐AKT signaling pathway was enriched among the cellular pathways, The PI3K‐AKT pathway was suppressed in NDHG‐ECs (Figure S11B) and activated in pEPCs (Figure 4B). This finding was further verified by western blot analysis (Figure 4C). These results indicate that the protective effect of sb‐NDHG preconditioning partly relies on the upregulation of the PI3K‐AKT signaling pathway in pEPCs.

To determine whether the activation of the PI3K‐AKT signaling pathway is essential for the protective effects of sb‐NDHG preconditioning, we pretreated ECs with the PI3K inhibitor LY294002 (LY) to block the PI3K‐AKT signaling. Following this treatment, the cells underwent sb‐NDHG preconditioning and referred to as LY+pECs. Compared to pEPCs and ECs, LY+pECs exhibited a reduced proliferation rate, lower CD34 expression (Figure 4D,E), as well as impaired tube formation (Figure 4F,G). Furthermore, according to the results of qRT‐PCR and western blot analysis, the expression levels of Nrf2 and SOD in LY+pECs were reduced, similar to those in NDHG‐ECs (Figure 4H,I). Collectively, these results suggest that sb‐NDHG preconditioning promotes the activation of PI3K‐AKT signaling pathway, thereby upregulating its downstream signal pathway, which appears to play a crucial role in the protective effects for pEPCs mediated by sb‐NDHG preconditioning.

### 2.5 pEPCs Exhibit Longer in Vivo Survival and Retention Than ECs in Diabetic Mice

**FIGURE 5:**
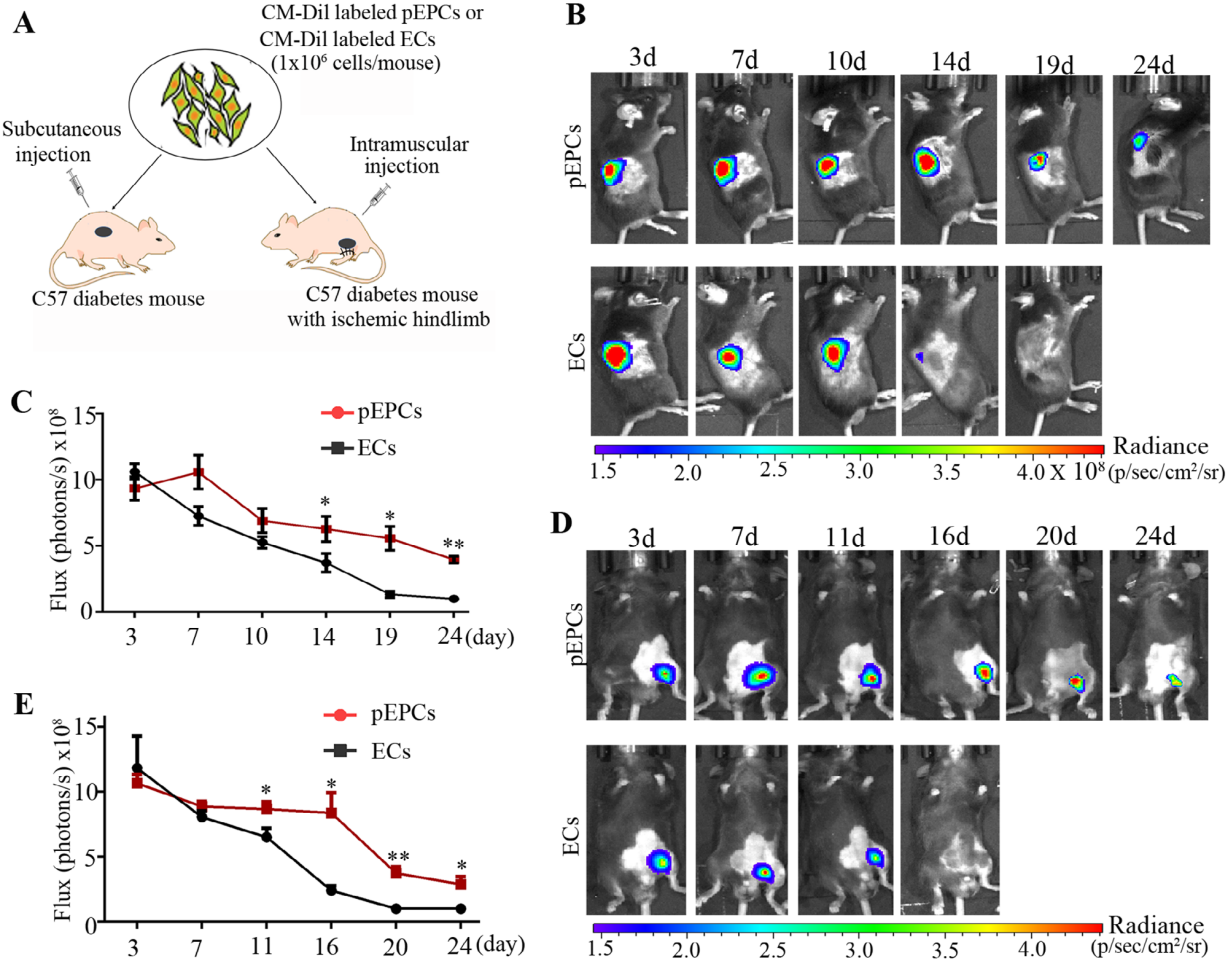
Evaluation of the survival/retention of pEPCs in diabetic mice. (A) A description of the experimental scheme. (B) CM‐Dil‐labeled pEPCs or CM‐Dil‐labeled ECs were subcutaneously injected into the dorsal skin of diabetic mice (*n* = 6). The fluorescence signal of each recipient mouse was measured at the indicated time points. (C) Quantitative analysis of the fluorescent signal in each recipient mouse over a 24‐day period. (D) CM‐Dil‐labeled pEPCs or CM‐Dil‐labeled ECs were intramuscularly injected into the ischemic hind limbs of diabetic mice (*n* = 6). Fluorescence signal of each recipient mouse was measured at the indicated times post‐cell injection. (E) Quantitative analysis of fluorescence signal in each recipient mouse over a 24‐day period. Data are represented as means ± SEM. *p < 0.05; **p < 0.01 compared with the corresponding control.

The survival and retention of cells in vivo are important indicators of their potential use and effectiveness in cell‐based therapy for a variety of refractory diseases, including DW [29, 30]. To assess the survival and retention of pEPCs in vivo, CM‐Dil‐labeled pEPCs were subcutaneously injected into the dorsal skin of diabetic mice. Mice injected with CM‐Dil‐labeled ECs served as controls (Figure 5A). Over an observation period of 24 days, we continuously monitored the fluorescence signals at the injection sites of the pEPCs. In contrast, the fluorescence signals from the ECs decreased dramatically over time and were almost undetectable by the 19th day (Figure 5B). Quantitative analysis of the fluorescence signals revealed a trend consistent with these observations (Figure 5C). Based on these results, we infer that pEPCs maintain stability for a longer duration than ECs in the non‐wounded skin of diabetic mice following administration.

To further assess whether pEPCs can survive in the areas of lesions, CM‐Dil‐labeled pEPCs were injected intramuscularly into the ischemic limbs of diabetic mice. CM‐Dil‐labeled ECs as controls (Figure 5A). The fluorescence signals from ECs remained relatively stable for about 7 days after injection, and then began to decrease exponentially, reaching baseline levels within 16–20 days. In contrast, the fluorescence signals of pEPCs persisted for a longer duration, remaining detectable up to 24 days post‐injection (Figure 5D,E). Biosafety of pEPCs is an important issue that requires attention. To assess the safety of pEPCs, pEPCs were transplanted into diabetic model mice for a duration of 3 months, in which that levels of alanine aminotransferase (ALT), aspartate aminotransferase (AST), and immunoglobulin G2 (IgG2) were not significantly different from those in normal mice, and no tumor‐like structures were observed in the organs of the model mice (Figure S12). Collectively, these findings demonstrate that pEPCs exhibit higher levels of survival and retention as well as biosafety in vivo.

### 2.6 pEPC Transplantation Enhances Blood Flow Recovery in Ischemic Limbs of Diabetic Mice

**FIGURE 6:**
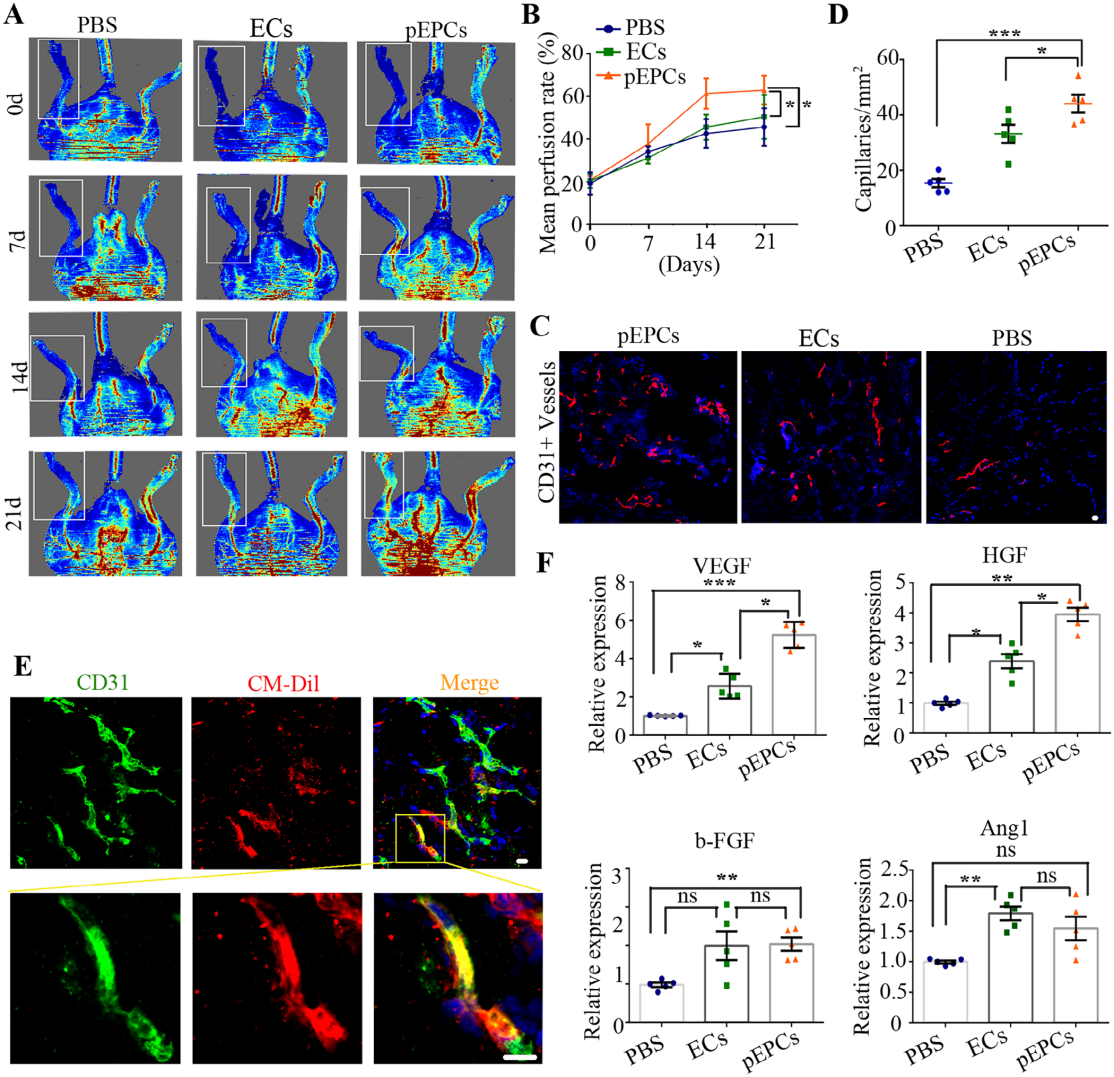
pEPCs enhance blood flow recovery in a diabetic mouse model of hindlimb ischemia. (A) Representative Doppler images of mice in different groups at 0, 7, 14, and 21 days post‐treatment (*n* = 6). The white square frame denotes the ischemic limbs. (B) Perfusion rates of ischemic limbs at days 0, 7, 14, and 21 after cell treatment. (C) Immunofluorescence staining for endothelial‐specific CD31 in ischemic limbs implanted with ECs, pEPCs, or PBS. (D) Quantification of vessel density and the percentage of CD31+ vessels. (E) Representative images showing the incorporation of pEPCs into the host microvasculature. (F) Expression of angiogenesis factors in ischemic muscle tissue was tested by qRT‐PCR. Data are presented as means ± SEM. *p < 0.05, **p < 0.01, ***p < 0.001 compared with the corresponding control. Scale bars represent 20 µm.

To assess the therapeutic efficacy of pEPC transplantation, we administered pEPCs or ECs intramuscularly into the ischemic hind limbs of diabetic mice that had been subjected to femoral artery ligation. Mice injected with an equivalent volume of PBS served as controls, and blood flow was monitored using laser Doppler imaging (Figure 6A). On the zeroth day, the average perfusion rate (PR) of blood flow in all mice that were treated by surgery was approximately 20.0%, confirmed that severe limb ischemia was successfully induced in the diabetic mice. Compared with the PBS‐treated and ECs‐treated mice, the average PR in pEPCs‐treated mice gradually increased and peaked 14 days post‐injection (Figure 6B), indicating that pEPCs significantly enhanced the recovery of blood flow in ischemic hindlimbs.

The improvement in limb perfusion was further confirmed with immunofluorescence quantification of total vascular density using the endothelia‐specific CD31 antibody (Figure 6C). Quantitative analysis showed that mice transplanted with pEPCs had a capillary density nearly three times higher than that of mice treated with PBS, and approximately 1.5 times higher than that of mice treated with ECs (Figure 6D). To verify the integration of pEPCs into the host vasculature, CM‐Dil‐labeled pEPCs were injected intramuscular into the ischemic limbs of diabetic mice. Alexa568‐isolectin‐B4 was perfused into the mice 14 days post pEPCs transplantation to identify all blood vessels. We observed that CM‐Dil+ human‐derived vessels were interspersed among the murine vessels in the ischemic limb. This finding indicates that pEPCs have the potential to incorporate with host microvasculature (Figure 6E).

Comprehensive expression of angiogenic growth factors is beneficial for promoting angiogenesis in ischemic limbs. Seven days after pEPCs transplantation, RNA was extracted from the limbs of each group. qRT‐PCR analysis revealed that in comparison with PBS‐treated group, the expression of the genes VE*GF, bFGF*, and *HGF* in the pEPCs‐treated group was significantly upregulated (Figure 6F). These results further confirm the excellent therapeutic effects of pEPCs in the treatment of DW.

### 2.7 Transplantation of pEPCs Enhances Neovascularization and Re‐Epithelialization in Diabetic Wound Healing

**FIGURE 7:**
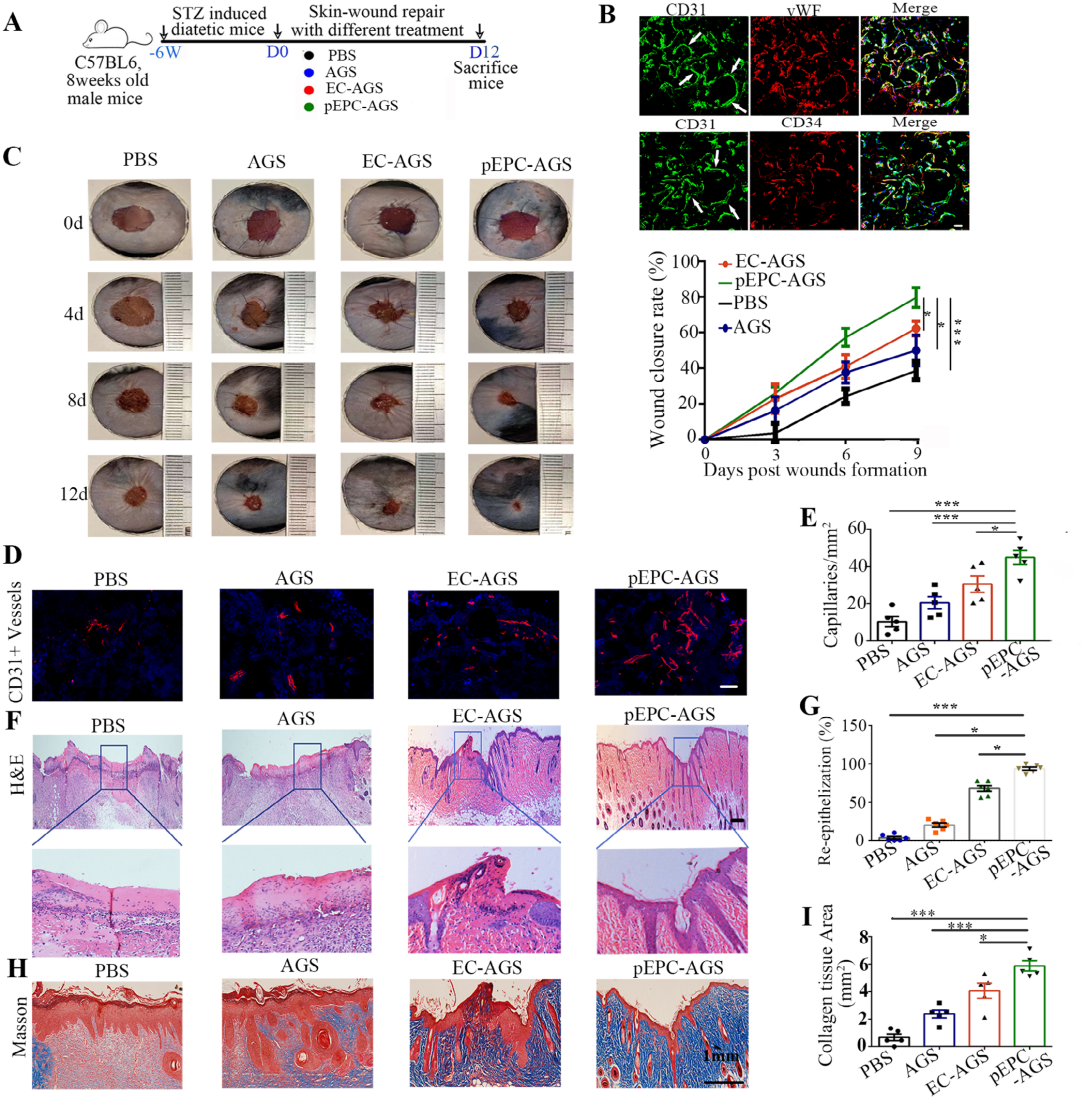
pEPCs accelerate wound healing in diabetic mice. (A) Illustration of the experimental protocol. (B) Immunostaining of endothelial markers in pEPCs cultured in the shaking bed for 3 days. White arrows indicate the typical tubular‐like structure formed by pEPCs in AGS. (C) Representative wound images at different time points post‐wounding (left) and quantification of wound closure rate (right) (*n* = 6). (D) Representative images of capillaries stained with CD31 antibody (red) in skin sections. (E) Quantification of the number of capillaries in five randomly selected areas of each skin sample. (F) Harvested skin tissues (12 days post injury) were cross‐sectioned to assess the degree of re‐epithelialization. High‐magnification views of the indicated regions are shown (bottom). (G) Quantification of re‐epithelialization of skin sections. (H) Evaluation of collagen deposition with Masson's trichrome dyes. (I) Quantification of collagen deposition. Data are presented as mean ± SEM. *p < 0.05, ***p < 0.001 compared with the corresponding control. Scale bars represent 50 µm in (B) and (D), 500 µm in (F), and 1 mm in (I). EC‐AGS, ECs‐loaded AGS; pEPC‐AGS, pEPCs‐loaded AGS.

To assess the therapeutic potential of pEPCs in DW healing, we created a 10‐mm full‐thickness skin wound on the dorsum of each diabetic mouse and then administered different treatments (Figure 7A). First, pEPCs and ECs were pre‐planted on an absorbable gelatin sponge (AGS), a material widely used in bone regeneration and nerve repair [31]. After 3–4 days of culture in a shaker, pEPCs proliferated within the AGS and formed typical tubular structure (Figure 7B). The mice successfully modeled were randomly divided into four groups, which received (1) pEPCs‐loaded AGS (pEPC‐AGS), (2) ECs‐loaded AGS (EC‐AGS), (3) pure AGS, or (4) PBS, respectively. The AGS was placed over the wound and affixed to the skin using interrupted 6–0 nylon sutures. The wounds were observed with digital photographs (Figure 7C, left). Quantitative analysis showed that the wound treated with pEPC‐AGS was approximately 64% closed after 9 days and nearly fully closed after 12 days (Figure 7C, right), indicating that pEPC‐AGS healed the wound much faster than other treatments.

Angiogenesis plays a crucial role in wound regeneration. The improvement of angiogenesis in DWs was further confirmed 12 days post‐wounding with vascular staining with a CD31 antibody (Figure 7D). Quantitative analysis of capillary density, which is defined as the number of CD31‐positive cells per square millimeter, showed that wounds transplanted with pEPCs exhibited a higher capillary density than other groups (Figure 7E).

H&E staining was performed to evaluate the degree of re‐epithelization (Figure 7F). Notably, by 12 days post‐wounding, the wounds treated with pEPC‐AGS exhibited intact newly formed epidermis and dermis, complete with hair follicles. In contrast, wounds treated with either PBS or AGS alone did not develop a new epidermis (Figure 7G). Higher magnification images clearly depicted the extent of re‐epithelization across the groups (Figure 7F). Masson's trichrome staining was utilized to detect collagen deposition, revealing a greater quantity of collagen fibers in wounds treated with pEPC‐AGS compared to other groups (Figure 7H). Quantification of the average intensity of Masson‐stained areas further confirmed the superior efficacy of pEPC‐AGS in promoting collagen deposition during wound healing (Figure 7I). These data indicate that the combination of pEPC with AGS accelerates cutaneous wound healing in diabetic mice.

## 3 Discussion

Cell therapy has provided a considerable opportunity for DW treatments, for which EPC‐based therapy is uniquely suited to, by allowing a more precise route to the area of ischemia, increase in vivo angiogenesis and the promotion of tissue repair [32, 33]. However, poor cell survival and low engraftment of EPCs at the harsh and hostile environment after transplantation limit their regeneration potential [9, 34]. Genetic modification and cellular reprogramming, as well as preconditioning, are strategies that could potentially optimize cell‐based therapy. Our study demonstrates for the first time that preconditioning of ECs under NDHG condition with sb431542 is required to induce the conversion of ECs into EPC‐like cells. These pEPCs acquire antioxidant capacity, maintain cell viability, and demonstrate stable tube formation ability in stressful environments. Importantly, pEPCs are more effective for blood vessel formation and tissue repair in diabetic mouse models. Collectively, this study provides a simple and effective method and can promote pEPC therapeutic functions to help to push them toward a more beneficial therapeutic phenotype for the treatment of DW.

Increasing evidence has shown that preconditioning, including exposure to hypoxia, pharmacological or chemical agents, growth factors, and hormones, can optimize the therapeutic efficacy of transplanted cells. Among them, hypoxic‐preconditioning is highly studied. In these studies, hypoxic‐preconditioned stem cells showed improved survival and enhanced differentiation. Indeed, the ischemic area is strongly hypoxic. When EPCs are exposed to hypoxia, cellular adaptation to harsh conditions is triggered through the upregulation of HIF‐1α, which would activate the HIF‐1a‐VEGF signal pathway and promote the secretion of angiogenic factors VEGF and bFGF. This alleviates endothelial injury caused by ischemic hypoxia in ischemic conditions, such as acute myocardial ischemia. However, in the DW microenvironment, EPCs gradually lose their protective regulatory function due to chronic hypoxia, resulting in insufficient secretion of angiogenic factors. Consequently, after EPCs transplantation, these cells encounter survival challenges due to not only elevated glucose levels but also reduced growth factors in the pathological microenvironment. To simulate the harsh microenvironment of DW, we replaced hypoxia with nutrient deprivation and high‐glucose conditions. In this study, ECs were preconditioned under sb‐NDHG condition, which promoted cellular adaptation to the harsh environment prior to in vivo transplantation by amplifying beneficial signaling pathways. The activation of the PI3K‐Nrf2/SOD signaling pathway in pEPCs improved stress tolerance and enhanced their therapeutic potential in vivo. This method aligns with the current popular method of cell preconditioning, also known as injury‐induced protection, indicating that NDHG conditions are a critical element in this preconditioning method.

In this study, we also found that inhibition of TGF‐β signaling plays a crucial role in this preconditioning method. Several studies have provided evidence that inhibition of TGF‐β signaling helps maintain the viability and proliferation capacity of ECs. Other studies have revealed that the activation of TGF‐β signaling favors hyperglycemia‐induced endothelial‐to‐mesenchymal transition as well as hyperglycemia‐induced EC metabolic memory [25, 35], which impedes the proliferation of ECs and abrogates their angiogenic function [20]. Our findings indicate that under NDHG conditions, TGF‐β signaling in ECs is activated, suggesting a correlation between the activation of TGF‐β signaling and the reduced activity of ECs. Based on previous research and our experimental results, sb431542, a common inhibitor of the TGF‐β signaling pathway, was selected to include in our preconditioning protocol. The results indicate that sb431542 can enhance the viability of ECs cultivated under NDHG conditions. Prolonging the culture time allowed the acclimated ECs to gradually increase CD34 expression, stabilize their tube formation capability, and transform into enhanced vasculogenic EPC‐like cells.

Conversely, if NDHG conditions are replaced with EC‐culture medium and only sb431542 is added, ECs will not significantly increase the expression of CD34 and improve their adaptability to NDHG conditions, even with prolonged culture time. The results indicate that the compound sb431542 is a key component in this preconditioning protocol and works synergistically with the NDHG condition to induce the formation of pEPCs.

In addition, some other small molecule compounds that target other signaling pathways, such as AMPK signaling, have been reported to protect against high glucose or oxidative stress‐induced endothelial dysfunction and are considered drug candidates for cardiovascular disorders [36]. In this study, three widely studied small molecule compounds, including SRT‐1460, MDL‐28170, and lovastatin, were chosen to examine whether they could exert effects similar to those of sb431542. Our observations reveal that none of them were effective in stabilizing EC viability under NDHG conditions, which makes it even less likely that they can enhance EC adaptability to harsh environments after preconditioning. These results confirm that the TGF‐β inhibitor sb431542 is effective and a vital part of our preconditioning method.

The primary challenge in cell‐based therapy is the extremely low rate of survival and retention of cells in the wound tissue, whereas the survival and retention of pEPCs were significantly increased than parental ECs, not only in the subcutaneous tissue but also in the ischemic limbs of diabetic mice, which is crucial for the success of cell therapy [29, 30, 37]. Moreover, the beneficial effects of pEPCs could be in part attributed to their ability to increase the paracrine factors in the bloodstream at the wound site, such as VEGF and bFGF, and their differentiation into vascular cells that integrate into the neovascular system. This study demonstrates that the positive effects of pEPCs are likely to be multi‐factorial.

With advancements in technology, several studies have explored new methods to generate autologous ECs from pluripotent stem cells [38, 39], or reprogrammed non‐endothelial cells [40, 41, 42]. These approaches offer the potential to obtain pEPCs. However, there is still a long road ahead, and further research is needed to address important aspects such as comparative studies of optimal delivery routes, determining the appropriate cell dosage, and exploring the combination of pEPCs with biomaterials or growth factors. These investigations are crucial to ensure the effectiveness and safety of pEPC transplantation in the future.

## 4 Conclusions

In summary, the present study provides a simple and effective preconditioning protocol for obtaining functionally enhanced pEPCs, and they hold promise as an alternative cell source for the treatment of diabetic ischemic diseases.

## 5 Materials and Methods

### 5.1 Cell Culture and Treatments

Human umbilical cord was obtained from health donors in West China Hospital, Sichuan University, upon consent of its donor according to procedures approved by the Medical Ethics Committee, Sichuan University. Human umbilical vein endothelial cells (hUVECs, abbreviated as ECs) were isolated as described and cultured with EGM, which is comprised of endothelial basal medium (EBM, ScienCell, 1001), 5% fetal bovine serum (FBS; ScienCell, 0025), and 1% endothelial cell growth supplements (ECGS; ScienCell, 1052). The nutrient‐deficient and high glucose‐containing (NDHG) conditions was used to simulate the pathological condition of DW. Specifically, extra 50 mM d‐glucose was added into EBM, which contained only 2% FBS and no ECGS.

To screen small molecule compounds, ECs were cultured under NDHG conditions, each supplemented with a different small molecule. Cell viability was evaluated using Cell Counting kit‐8 (CCK8), TUNEL staining, flow cytometry, and live/dead staining according to the manufacturer's instructions. Important information about small molecule compounds is listed in Table S1.

### 5.2 In Vitro Endothelial Functional Assays

The uptake of Dil‐acLDL was assessed by incubating cells with 10 µg/mL of ac‐LDL (Invitrogen) for 4 h before detection. For Matrigel tube–like formation in vitro assay, pECs at passages 1, 2, and 3 were trypsinized into single cells. ECs cultured in EGM were used as controls. The resuspended cells (5 × 10^4^/100 µL) were seeded on top of a thin layer of Matrigel with either EGM or NDHG conditions. The formation of tube‐like structures was imaged under brightfield microscopy at the indicated time points. The total tube length and the number of loops were counted and quantified.

### 5.3 Flow Cytometry Analysis

To detect cells phenotypes, pECs and ECs were trypsinized into single cells and incubated with fluorescence‐labeling mAbs for 30 min on ice in the dark. The fluorescence intensity was measured using a flow cytometer (BD Biosciences) and analyzed using FlowJo software (Treestar, Ashland, OR, USA). The antibodies are listed in Table S2.

To detect the level of intracellular ROS, pECs, ECs, and NDHG‐ECs (ECs that were cultured under NDHG conditions) were collected and incubated with 10 µM H2DCF‐DA (Beyotime, China) for 20 min at 37°C in dark. After washing with cold PBS, the samples were analyzed using a flow cytometer. The level of intracellular ROS was calculated based on the mean cell fluorescence intensity. To evaluate cellular apoptosis rate, the dissociated cells that cultured in different conditions were incubated with Annexin V‐FITC apoptosis detection kit (Beyotime, China) for 10 min in dark at room temperature (RT), and then analyzed using a flow cytometer.

### 5.4 Immunofluorescence and Histological Analyses

For immunofluorescence staining, cells fixed with 4% PFA were blocked with normal goat serum (NGS, ThermoFisher Scientific) for 30 min at RT, and subsequently stained with primary monoclonal antibodies (mAbs) at 4°C overnight. After thorough washing with PBS, the cells were incubated with secondary mAbs for 1 h at RT in the dark. Cellular nuclei were stained with DAPI (Roche Basel, Switzerland). For blood vessels immunostaining, O.C.T‐embedded tissue was cut into 10‐µm‐thick sections. Sections were then fixed with ice‐cold acetone for 10 min before being stained with the primary and secondary mAbs. The sections were visualized using a laser scanning confocal microscope. The antibodies used are listed in Table S2.

### 5.5 Western Blot Analysis

Western blot analysis was used to detect the expression of specific proteins in cells following different treatments, according to standard procedures. Protein concentrations were measured using a BCA protein assay kit (Beyotime). A total of 30 µg of protein was separated using a 12% SDS‐PAGE gel, then transferred to nitrocellulose membranes. After blocking with 5% fat‐free milk for 2 h, the membranes were incubated with primary antibodies at 4°C overnight, followed by incubation with an HRP‐conjugated secondary antibody (GE Healthcare, Buckinghamshire, UK). GAPDH mAb was used as an internal control. The primary and secondary antibodies are listed in Table S2.

### 5.6 RNA‐Seq and Quantitative Real‐time PCR

Total RNAs was extracted from cells and sequenced using the Solexa high‐throughput sequencing service (Majorbio, China). The RNA‐seq raw expression files and details have been deposited in NCBI GEO under accession nos. SRR22198247, SRR22198248, and SRR22198249. The RNA was reverse transcribed with M‐MLV Reverse Transcriptase (Takara). Quantitative real‐time PCR (qRT‐PCR) was performed using SYBR Green PCR Master Mix (Takara). Gene expression was normalized to β‐actin. Fold changes in the expression levels of target samples relative to control samples were calculated using the 2^−ΔΔ^CT method. The primers used are provided in Table S3.

### 5.7 Diabetic‐Hindlimb Ischemia Models and Assessments

Male C57BL/6J mice were purchased from Beijing Huafukang Bioscience. Animal experiments were performed in accordance with the protocols approved by the Animal Care and Use Committee of Sichuan University. Experimental Diabetes Mellitus was induced in 8‐week‐old mice by daily injection of streptozotocin (STZ, 70 mg/kg, Amresco, USA) for five consecutive days, following a 4‐week high‐fat diet regimen. Blood glucose levels were monitored after 4–5 weeks of STZ administration. Mice with sustained blood glucose levels above 250 mg/dL were selected and defined as STZ‐induced diabetic mice.

Hind limb ischemia was induced by sequential ligation and excision of the femoral arteries, **the femoral artery spans** from its proximal origin at the branch of the external iliac artery to the distal point where it bifurcates into the saphenous and popliteal arteries [5]. Right after arterial excision, 1.0 × 10^6^ ECs or pEPCs (suspended in 150 µL PBS) were injected into ischemic limbs at three equally spaced points. Cyclosporine A was added to the drinking water at a concentration of 210 mg/L, during the experimental period. The blood flow was tested weekly using the laser Doppler imaging (Laser Doppler Perfusion Imager System, Moor Instruments) for 3 weeks. The recovery of the perfusion ratio (PR) was calculated as the ratio of ischemic to non‐ischemic hind limb blood flow.

To assess capillary perfusion, tissue sections from mice muscle were stained with human/mouse CD31 antibody. The number of capillaries in five random microscopic fields was counted and expressed as the number of capillaries per square millimeter of tissue. To test whether pEPCs could fusion into the host vascular system, the transplanted pEPCs were pre‐labeled with cell‐tracker CM‐Dil (Invitrogen, C7000). Fourteen days after cell transplantation, the mice were perfused with Alexa568‐Isolectin‐B4 to identify functional vessels. The ischemic limbs that received cell transplantation were dissected for cryosectioning and detected with fluorescence microscopy.

### 5.8 Diabetic Wound Assay

A full‐thickness wound, 10 mm in diameter, extending through the panniculus carnosus, was created on the dorsum of the diabetic mice following an established model [43]. Before transplantation, ECs or pEPCs were pre‐seeded onto an AGS and cultured on a shaking bed for 3–4 days. The mice were randomized into four groups, wounds were covered with ECs‐AGS, pEPCs‐AGS, or pure AGS, and then affixed to the skin using 6‐0 nylon sutures (Ethicon, Somerville, NJ). Mice received a 150 µL PBS injection directly into the subcutaneous layer adjacent to the wound bed as negative controls. Photographs of the wounds were taken during the experimental period. Wound area was quantitated using ImageJ software, and wound closure rates were calculated using the following formula: wound closure rate (%) = (So − St) × 100%/So. So represents the initial wound area, and St represents wound area measured at days 0, 4, 8, and 12 post‐treatment.

### 5.9 In Vivo Imaging

To assess the survival of pEPCs in diabetic mice, CM‐Dil‐labeled pEPCs or CM‐Dil‐labeled ECs were injected into the ischemic limbs or non‐wounded dorsum skin of diabetic mice (a single injection of 1 × 10^6^ cells/mouse). Fluorescent signals were detected at the indicated time points using IVIS Spectrum imaging system (Perkin Elmer, Waltham, MA). The images were obtained and analyzed, and the signal intensity was represented by the radiance unit of photons (p)/s/cm^2^/sr.

### 5.10 Statistical Analysis

For statistical analysis of our data, GraphPad Prism software (GraphPad Software Ltd., San Diego, CA, USA) was used. Each experiment was performed a minimum of three times. Results were presented as means ± standard errors of the mean. A Student's *t* test was conducted to detect statistical difference between two groups. one‐way ANOVA with Bonferroni's post hoc analysis was performed to detect statistical difference among three or more groups. The results were considered statistically significant when *p* < 0.05.

## Author Contributions

D. S. S. and F. Y. C. were involved in the conception and design, collection and assembly of data, data analysis and interpretation, and manuscript writing. Y. Z. performed GO and KEGG analysis. Q. Y. J. and F. D. contributed to cell isolation and characterization, provision of study material, and assembly of data. C. P., Y. X. Y., and L. Z. were involved in the animal experiments. P. S. Z., H. L. W., Q. X., and X. L. S. were responsible for manuscript writing and revision of the manuscript. H. X. D. were involved in the conception and design, manuscript writing, financial support, and final approval of manuscript. All authors have read and approved the final manuscript.

## Ethics Statement

Human tissue was obtained according to procedures approved by the Medical Ethics Committee, Sichuan University (the registration number K‐2018109‐1), and written informed consent was obtained from all participants. All animal experiments conducted in our study have been approved by the appropriate ethical committee, with the approval number 2021366A.

## Conflicts of Interest

The authors declare no conflicts of interest.

## Supporting information




**Supporting Fig 1**: NDHG alone is not a suitable preconditioning condition for ECs.
**Supporting Fig 2**: Candidate small molecule compounds have limited effects on the reduction of cell apoptosis.
**Supporting Fig 3**: The optimum working concentration of sb431542 was determined.
**Supporting Fig 4**: The detection of TGF‐β expression in endothelial cells under different conditions.
**Supporting Fig 5**: The TGF‐β signaling pathway plays a crucial role in EPC preconditioning under NDHG conditions.
**Supporting Fig 6**: Other inhibitors targeting TGF‐β pathway can effectively reduce cell apoptosis
**Supporting Fig 7**: Long‐term preconditioning improves the stability of tube‐like structure in pECs
**Supporting Fig 8**: pEPCs were expanded in large quantities in vitro with stable biological characteristics
**Supporting Fig 9**: The detection of pro‐angiogenic cytokines.
**Supporting Fig 10**: pEPCs maintain stable biological properties after thawing from cryopreservation.
**Supporting Fig 11**: Hierarchical clustering and KEGG pathway enrichment analysis were performed on the RNA‐seq data for differentially expressed genes (DEGs) in the indicated cells.
**Supporting Fig 12**: Assessment of the Biosafety of pEPC.
**Supporting Table 1**: The list of small 218 molecule compounds.
**Supporting Table 2**: The list of antibodies.
**Supporting Table 3**: The list of PCR primer sequences.

## Data Availability

The data supporting the results in this study are available within the paper and its Supporting Information. The raw RNA sequencing data have been archived in the Gene Expression Omnibus repository under the accession number SRR22198247, SRR22198248, and SRR22198249, and any additional information required to reanalyze the data reported in this paper is available from the lead contact upon request.
