# Supplementary material for: Preconditioning With TGF‐β Inhibitors Enhances Therapeutic Efficacy of Endothelial Progenitor Cells for Wound Healing in Diabetic Mice
Source: MedComm (2020). 2025 Sep 1;6(9):e70364. doi: 10.1002/mco2.70364 (PMC12402589; doi:10.1002/mco2.70364)
Supplement: Supplementary file 1 — Supporting Fig 1: NDHG alone is not a suitable preconditioning condition for ECs. Supporting Fig 2: Candidate small molecule compounds have limited effects on the reduction of cell apoptosis. Supporting Fig 3: The optimum working concentration of sb431542 was determined. Supporting Fig 4: The detection of TGF‐β expression in endothelial cells under different conditions. Supporting Fig 5: The TGF‐β signaling pathway plays a crucial role in EPC preconditioning under NDHG conditions. Supporting Fig 6: Other inhibitors targeting TGF‐β pathway can effectively reduce cell apoptosis Supporting Fig 7: Long‐term preconditioning improves the stability of tube‐like structure in pECs Supporting Fig 8: pEPCs were expanded in large quantities in vitro with stable biological characteristics Supporting Fig 9: The detection of pro‐angiogenic cytokines. Supporting Fig 10: pEPCs maintain stable biological properties after thawing from cryopreservation. Supporting Fig 11: Hierarchical clustering and KEGG pathway enrichment analysis were performed on the RNA‐seq data for differentially expressed genes (DEGs) in the indicated cells. Supporting Fig 12: Assessment of the Biosafety of pEPC. Supporting Table 1: The list of small 218 molecule compounds. Supporting Table 2: The list of antibodies. Supporting Table 3: The list of PCR primer sequences. [file MCO2-6-e70364-s001.pdf]

Supplementary Information

Preconditioning with TGF-β Inhibitors enhances therapeutic efficacy of endothelial progenitor cells (EPCs) for Wound Healing in Diabetic mice

Dongsheng Su[1#], Fuyi Cheng[1,2#], Qingyuan Jiang[3#], Yong Zhang[1], Fei Du[1], Cheng Pan[2], Yixin Ye[1], Lin Zhang[1], Pusong Zhao[1], Huilin Wang[1], Qi Xiong[1], Xiaolan Su[1], Hongxin Deng[1*]

Correspondence: Hongxin Deng (denghongx@scu.edu.cn)

† Dongsheng Su, Fuyi Cheng, Qingyuan Jiang contributed equally to this work.

[1] Department of Biotherapy, Cancer Center and State Key Laboratory of Biotherapy， West China Hospital, Sichuan University, Chengdu, Sichuan, 610041, the people’s Republic of China.

[2] Department of Plastic, Reconstructive and Aesthetic Surgery, West China second university hospital, Sichuan University/West China Women’s and Children’s Hospital. Chengdu, Sichuan, 610041, the people’s Republic of China.

[3] Department of Obstetrics, Sichuan Provincial Hospital for Women and Children, Chengdu, Sichuan, 610041, the people’s Republic of China.

This PDF file includes:

1. Supplementary Figures and Figure Legends

2. Supplementary Tables

Figure S1

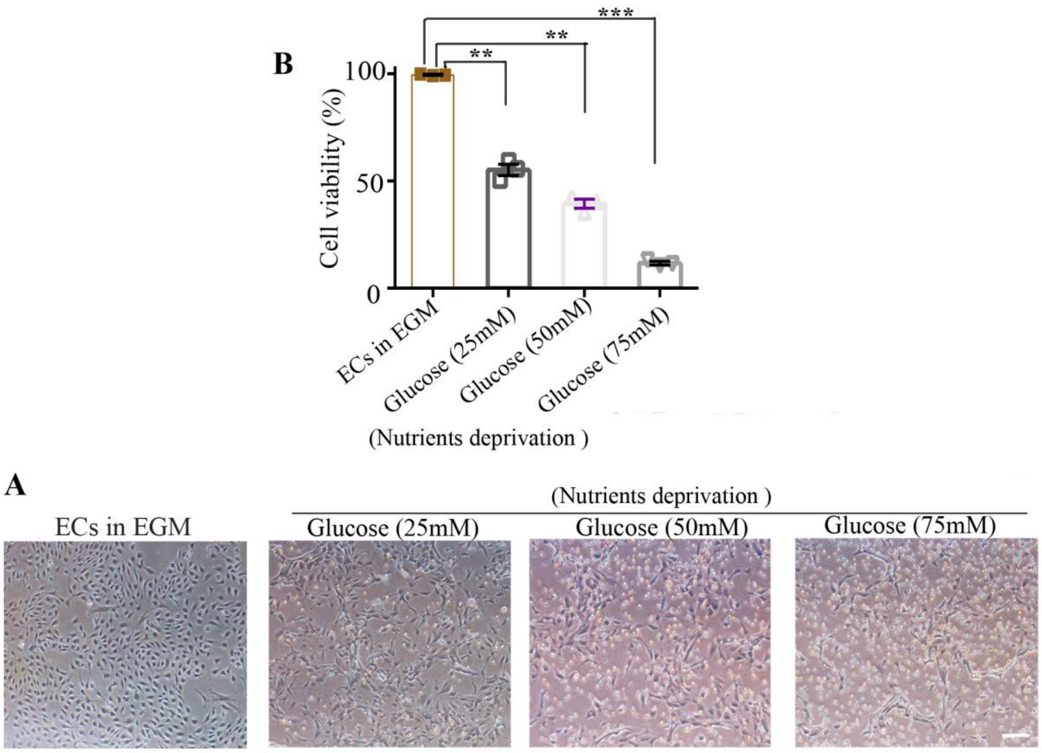

Figure S1. NDHG alone is not a suitable preconditioning condition for ECs. (A) Representative brightfield images of ECs cultured in NDHG conditions with various concentrations of D-glucose. ECs cultured in EGM served as control. (B) Quantification of cell viability based on the results of trypan blue staining among different groups. Data are represented as mean ± SEM. $^{**}p < 0.01$, $^{***}P < 0.001$. Scale bars represent 50 μm. Nutrients deprivation: shortage of endothelial growth factors in culture medium; EGM, endothelial growth medium.

Figure S2

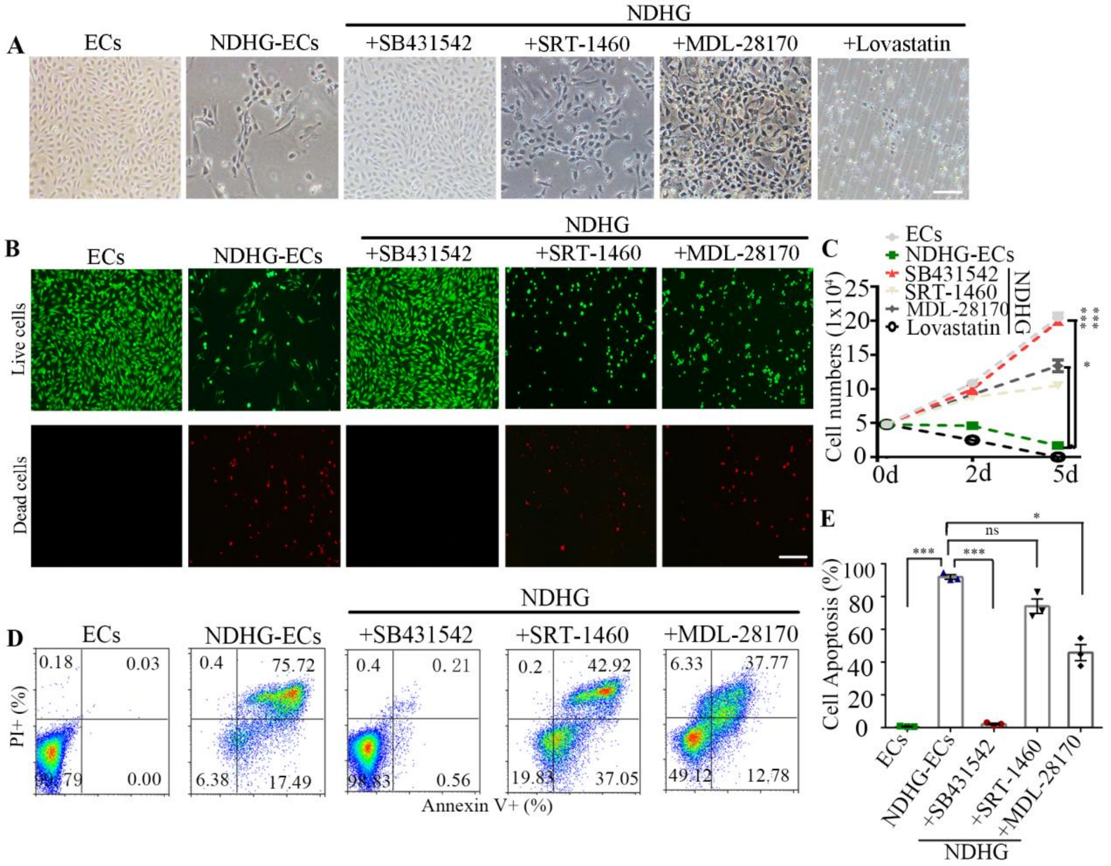

Figure S2. Candidate small molecule compounds have limited effects on the reduction of cell apoptosis. (A) ECs were cultured in NDHG with SB431542, SRT-1460, MDL-28170 and Lovastatin for five days. Representative brightfield images of cells are shown among different groups. (B) Cell viability was evaluated using live/dead fluorescence staining. (C) Quantification of cell viability was performed by counting the numbers of cell over five days. (D) Cell apoptosis was tested by flow cytometry. (E) Quantification of cell apoptosis based on the results of flow cytometry analysis. Data are represented as mean ± SEM. **p < 0.01, ***P < 0.001. Scale bars represent 50 μm.

Figure S3

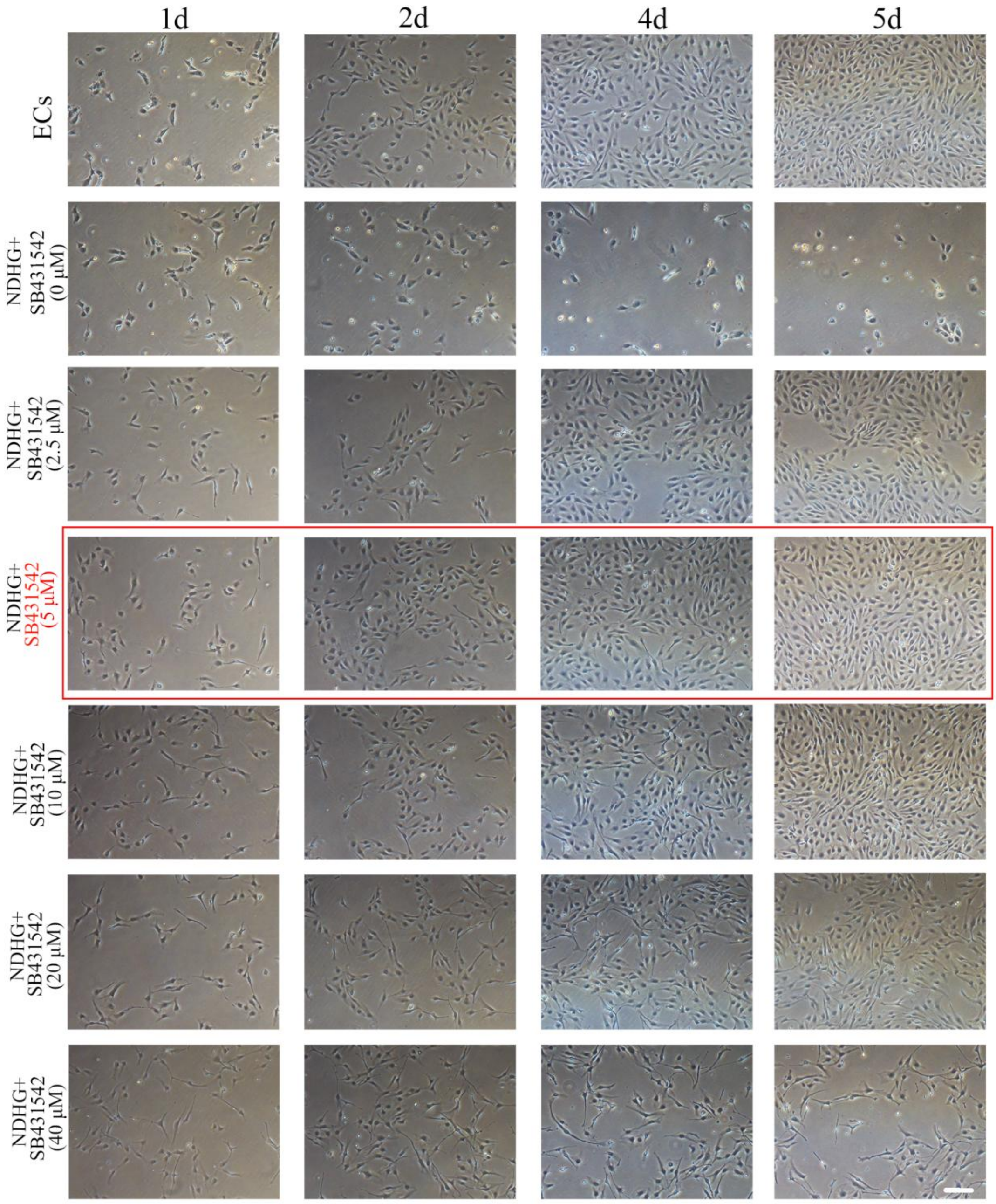

Figure S3. The optimum working concentration of sb431542 was determined. ECs were cultured in NDHG containing various concentrations of SB431542. Representative brightfield images of cells, cultured in different conditions at different times are shown. Scale bars represent 50 μm.

Figure S4

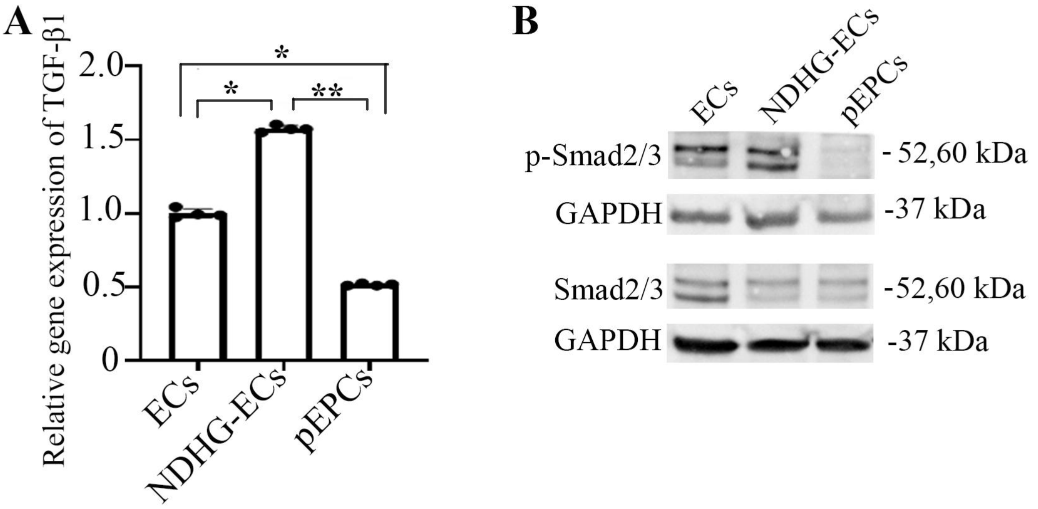

Figure S4. The detection of TGF-β expression in endothelial cells under different conditions. (A) mRNA levels of TGF-β1 were tested by qRT-PCR. (B) Protein levels of Smad2/3 and p-Smad2/3 were determined by western blot analysis. Data are represented as mean ± SEM. *$P < 0.05$, **$P < 0.01$. Three independent experiments were analyzed.

Figure S5

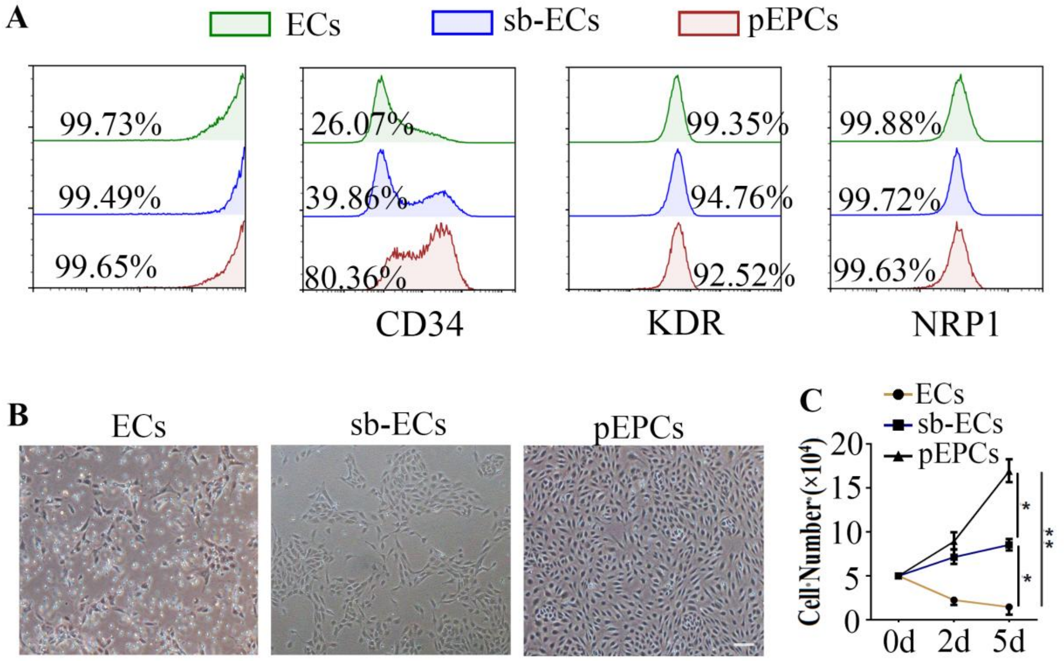

Figure S5. The TGF-β signaling pathway plays a crucial role in EPC preconditioning under NDHG conditions. (A) Endothelial surface markers were detected using flow cytometry analysis. (B) Representative brightfield images of the indicated cells cultured under NDHG conditions are shown. (C) Growth curves illustrate the number of cells counted per well at different time points. Data are represented as mean ± SEM. *P < 0.05, **P < 0.01. Three independent experiments were analyzed. Scale bars represent 50 μm.

Figure S6

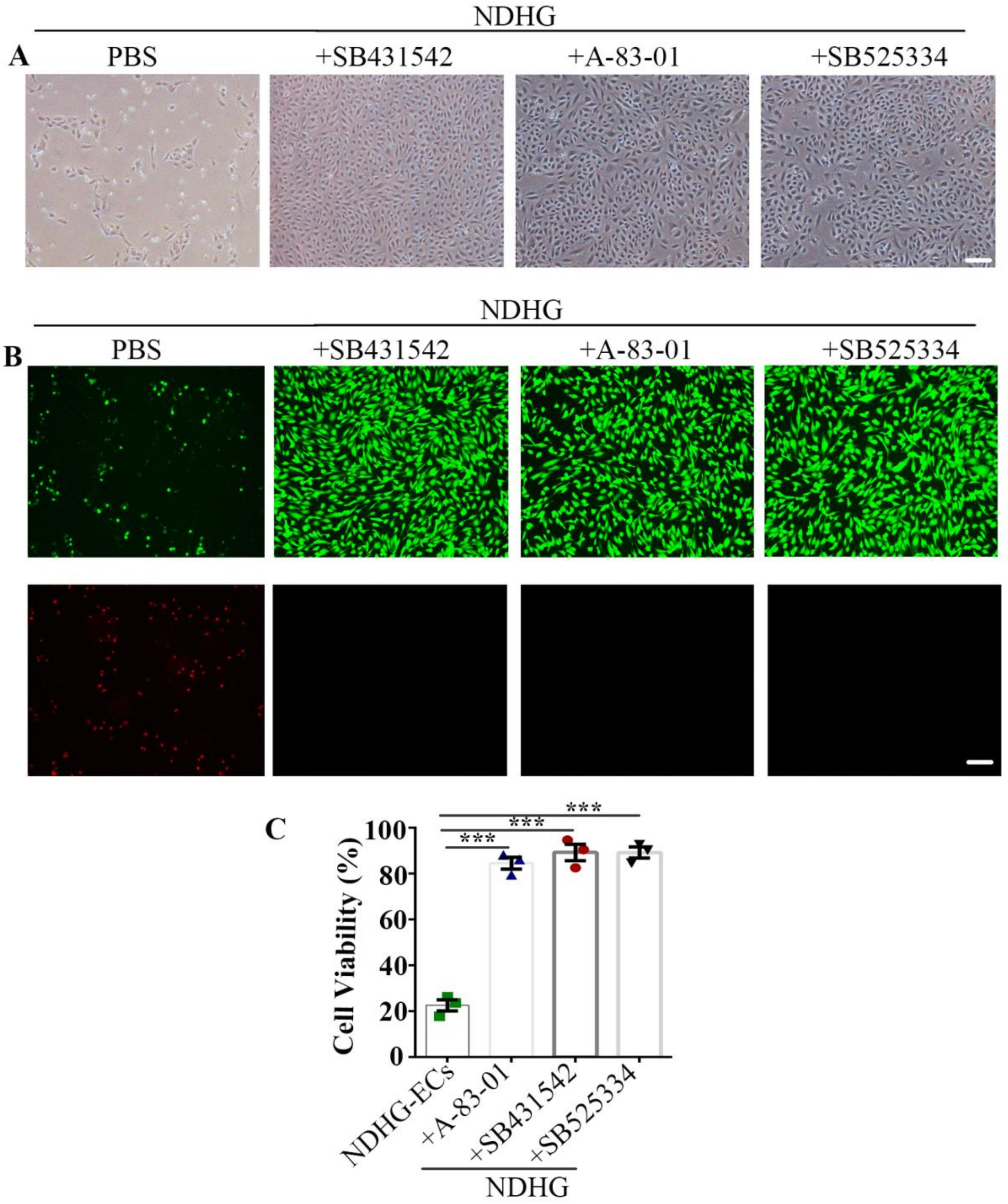

Figure S6. Other inhibitors targeting TGF-β pathway can effectively reduce cell apoptosis. (A) Representative bright-field images of ECs cultured in NDHG containing SB431542, SB 525334 or A-83-01 for three days are shown. (B) Cell viability was evaluated using live/dead fluorescence staining. (C) Quantification of cell viability was based on the results of live/dead fluorescent staining. Scale bars represent 50 μm. Data are represented as mean ± SEM, ***$P < 0.001$.

Figure S7

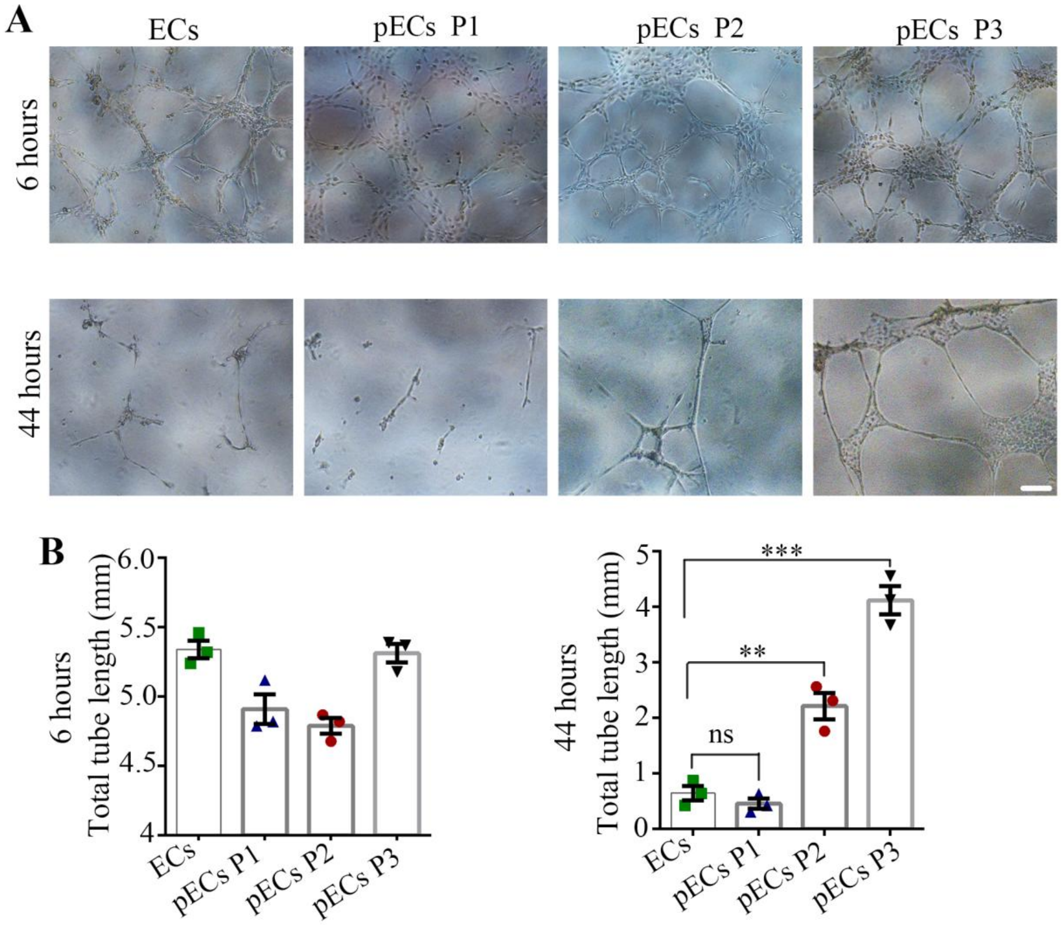

Figure S7. Long-term preconditioning improves the stability of tube-like structure in pECs. Preconditioned ECs at passages 1, 2 and 3 were harvested and then plated on Matrigel-coated plates in ECM medium. (A) Representative brightfield images of tube-like structure are shown after cell seeding for 6 hours and for 44 hours. (B) Tube lengths were measured 6 hours and 44 hours after seeding in Matrigel-coated plates. Data are represented as mean ± SEM. *P < 0.05, **P < 0.01, ***P < 0.001. Scale bars represent 50 μm.

Figure S8

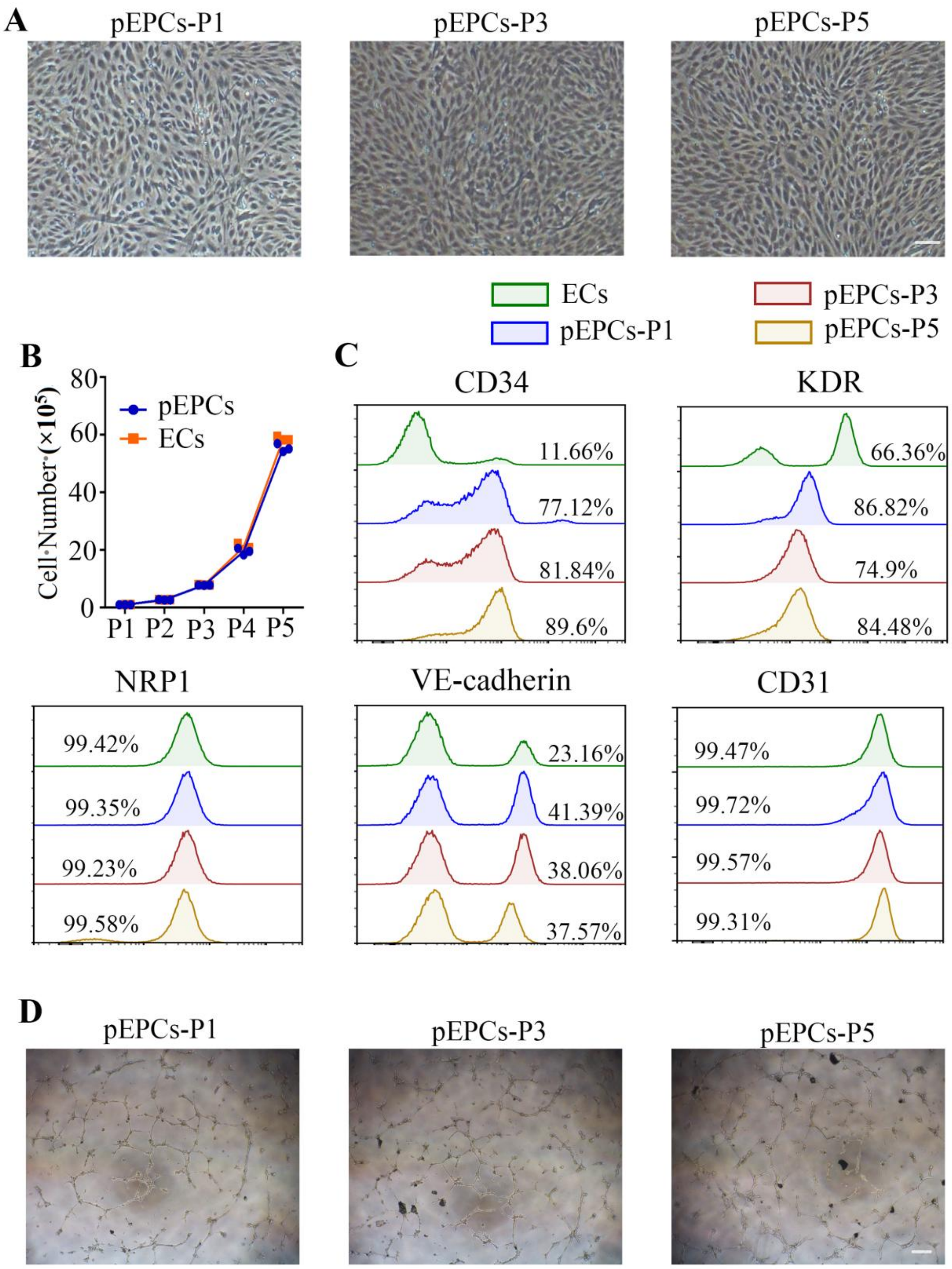

Figure S8. pEPCs were expanded in large quantities in vitro with stable biological characteristics. pEPCs cultured under sb-NDHG conditions were harvested for testing at different passages: P1, P3, and P5. (A) Representative brightfield images of pEPCs at passages P1, P3, and P5 are shown. (B) Growth curves illustrate the number of cells counted per well at different cell passages. (C) Endothelial surface markers were detected using flow cytometry analysis. (D) The

tube formation ability of the indicated cells was evaluated under NDHG conditions. Scale bars represent 50 μm.

Figure S9

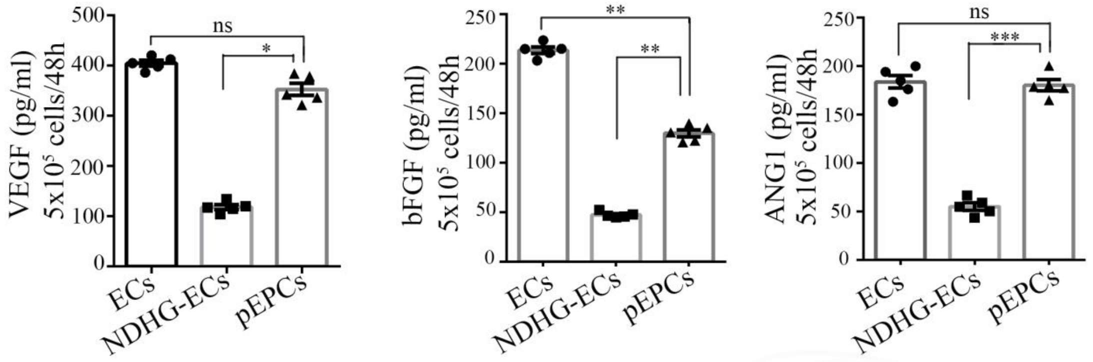

Figure S9. The detection of pro-angiogenic cytokines. Pro-angiogenic cytokines, including VEGF, bFGF and Ang1 were detected in the indicated cells using ELISA assays. Data are represented as mean ± SEM. *P < 0.05, **P < 0.01, ***P < 0.001. Three independent experiments were analyzed.

Figure S10

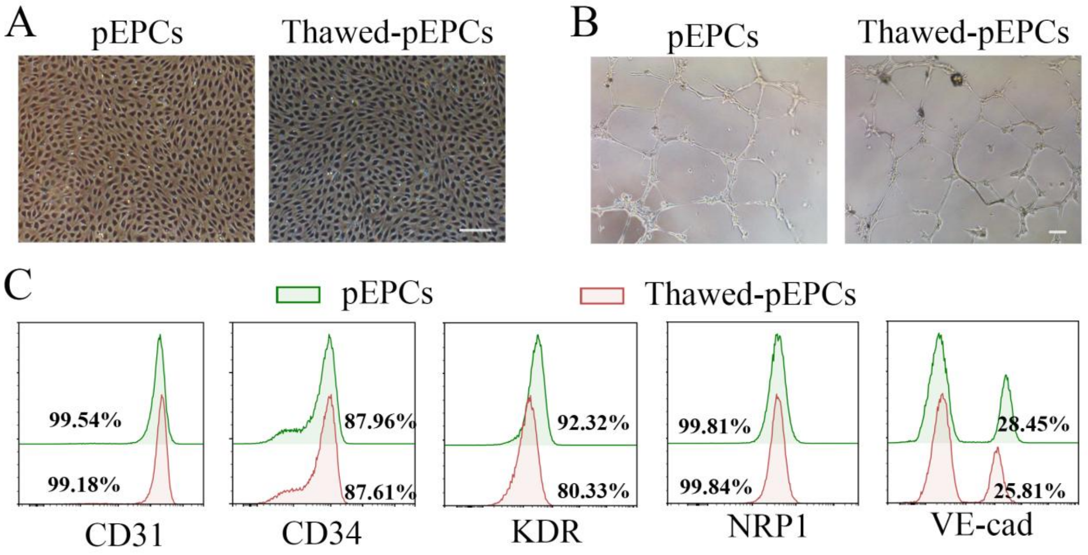

Figure S10. pEPCs maintain stable biological properties after thawing from cryopreservation. (A) Representative brightfield images of pEPCs and the thawed pEPCs are

shown. (B) The tube formation ability of the indicated cells was evaluated under NDHG conditions. (C) Endothelial surface markers were detected using flow cytometry analysis. Scale bars represent 50 μm.

Figure S11

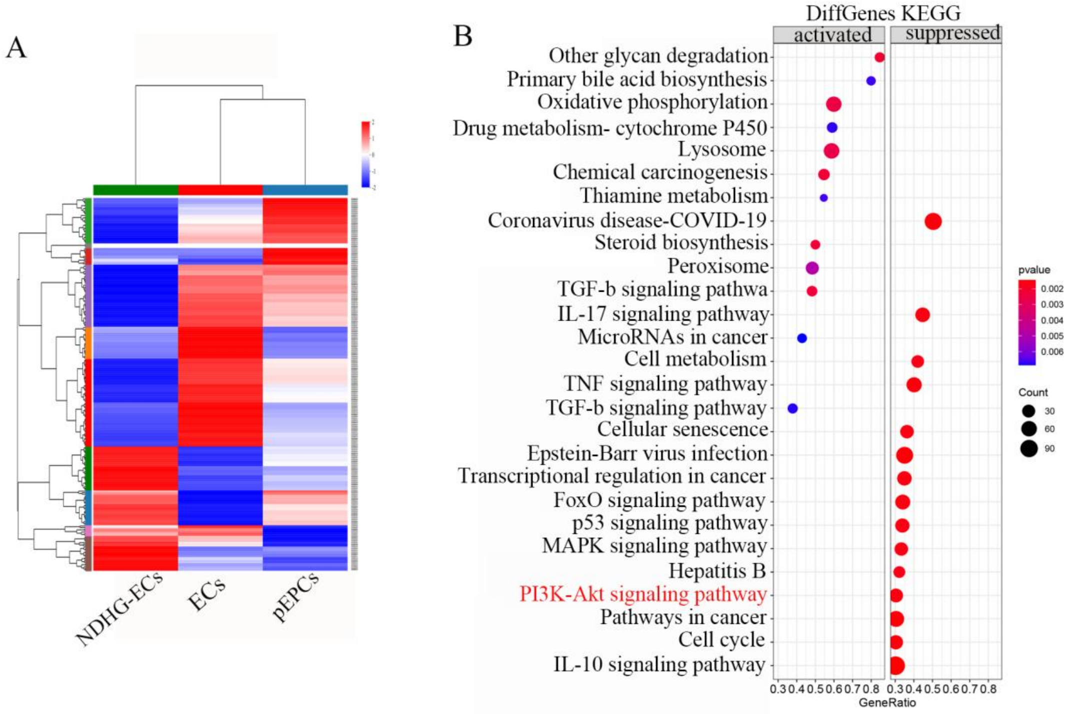

Figure S11. Hierarchical clustering and KEGG pathway enrichment analysis were performed on the RNA-seq data for differentially expressed genes (DEGs) in the indicated cells. (A) Heat map of the DEGs was created with cut-offs of $p<0.05$ and fold change >2. (B) KEGG pathway enrichment analysis was conducted between NDHG-ECs and pEPCs.

Figure S12

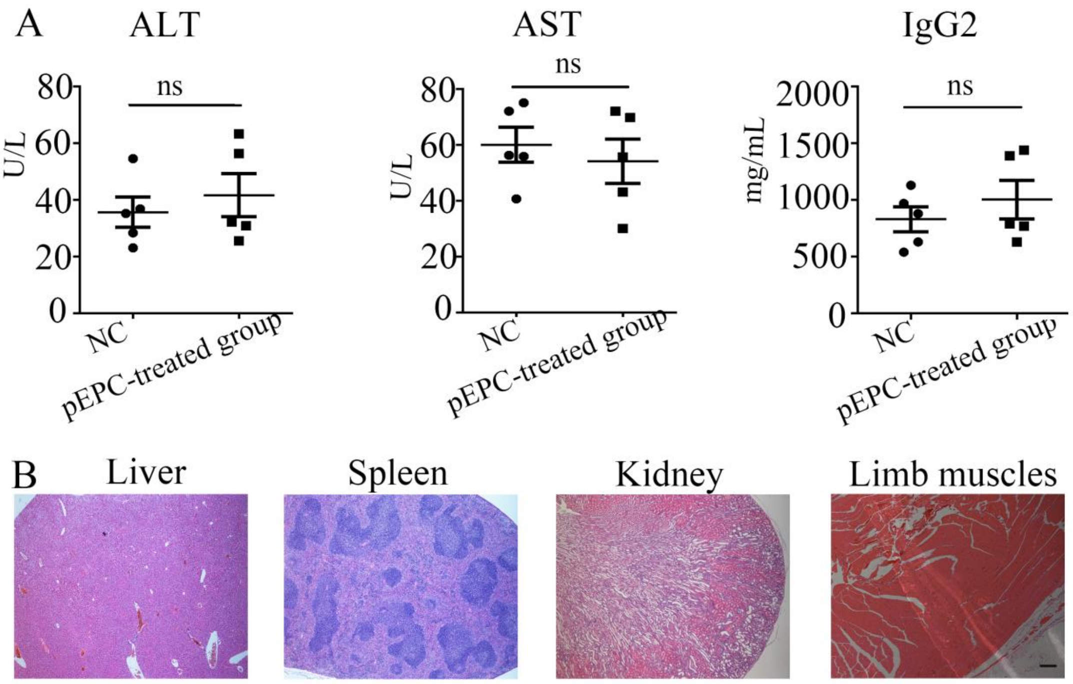

Figure S12. Assessment of the Biosafety of pEPC. (A) The levels of ALT, AST and IgG2 were tested by Elisa assays. (B) H&E staining was used to assess the tumorigenicity of cells in different tissues. Data are represented as mean ± SEM. Three independent experiments were analyzed. NC: Normal mice, pEPC-treated group: Mice that received pEPC transplantation. Scale bars represent 500 μm.

Table S1**: The list of small molecule compounds**

Product	Source	Catalog#	Target/ Signaling	The final working concentration
SB431542	Selleck	S1067	TGF-β	5 μM
A-83-01	Selleck	S7692	TGF-β	2.5 μM
SB525334	Selleck	S1476	TGF-β	5 μM
LY294002	Selleck	S1105	PI3K-AKT	10 μM
Lovastatin	Apexbio	A4365	GTP-Cyclohydrolase 1	10 μM
SRT-1460	Selleck	S6880	SIRT1 activator	2.5 μM
MDL-28170	Apexbio	A4412	Calpain inhibitor	2.5 μM

Table S2**: The list of antibodies**

Antibody	Source	Catalog#	Pur-pose	Host Specied & Reactivity	Concentration
DAPI	Roche	10236276	IF	Nucleus	1:1000
CD31	Abcam	Ab222783	IF	Mouse anti-human	1:200
CD31	Abcam	ab281583	IF	Rabbit anti-human and mouse	1:100
VE-cad	Abcam	ab33168	IF	Rabbit anti-human and mouse	1:200
vWF	Abcam	ab154193	IF	Rabbit anti-human	1:250
CD34	Abcam	ab81289	IF	Rabbit anti-human and mouse	1:200
Tunel	Beyotime	C1088	IF	Nucleus	1:200
CD31	Biolegend	303105	FC	Mouse anti-human	1x10^{6} cells/ 1.0 μg
CD34	Biolegend	343605	FC	Mouse anti-human	1x10^{6} cells/ 1.0 μg
KDR	Biolegend	359916	FC	Mouse anti-human	1x10^{6} cells/ 1.0 μg
NRP1	Biolegend	354508	FC	Mouse anti-human	1x10^{6} cells/ 1.0 μg
VE-cad	Biolegend	348506	FC	Mouse anti-human	1x10^{6} cells/ 1.0 μg
CD133	Biolegend	393903	FC	Mouse anti-human	1x10^{6} cells/ 1.0 μg
Annexin V-FITC Apoptosis Detection Kit	Beyotime	C1062M	FC	Cytomembrane	
Nrf2	Protein-tech	66504-1-Ig	WB	Rabbit anti-human and mouse	1:1000
SOD	Protein-	24127-1-	WB	Mouse anti-human	1:1000

	tech	AP			
GAPDH	HUA BIO	ET1601-4	WB	Rabbit anti-Human/Mouse	1:1000
PI3K	Affinity	AF6241	WB	Rabbit anti-Human/Mouse/Rat	1:1000
P-PI3K	Affinity	AF3241	WB	Rabbit anti-Human/Mouse	1:1000
AKT	CST	9272	WB	Rabbit anti-Human/Mouse	1:1000
P-AKT	CST	4058	WB	Rabbit anti-Human/Mouse	1:1000

Antibodies used in the study. IF= immunofluorescence; FC= flow cytometry; WB=Western Blot

Table S3**: The list of PCR primer sequences**

Gene	Sequence	
TGFB1	Forward	GGCCAGATCCTGTCCAAGC
	Reverse	GTGGGTTTCCACCATTAGCAC
ZEB	Forward	GCTGAGGATGACGGTATTGCCAA
	Reverse	GACTGCATGACCATCGCGTTCCT
Snail	Forward	TCGGAAGCCTAACTACAGCGA
	Reverse	AGATGAGCATTGGCAGCGAG
Slug	Forward	CGAACTGGACACACATACAGTG
	Reverse	CTGAGGATCTCTGGTTGTGGT
COLL-1	Forward	GAGGGCCAAGACGAAGACATC
	Reverse	CAGATCACGTCATCGCACAAC
a-SMA	Forward	GTGTTGCCCCTGAAGAGCAT
	Reverse	GCTGGGACATTGAAAGTCTCA
Nrf2	Forward	TCAGCGACGGAAAGAGTATGA
	Reverse	CCACTGGTTTCTGACTGGATGT
SOD1	Forward	AGGTCCTCACTTTAATCCTCTATC
	Reverse	CCAGCGTTTCCTGTCTTTGTAC
VEGF	Forward	AGGGCAGAATCATCACGAAGT
	Reverse	AGGGTCTCGATTGGATGGCA
b-FGF	Forward	AGAAGAGCGACCCTCACATCA
	Reverse	CGGTTAGCACACACTCCTTTG
ANG1	Forward	AGCGCCGAAGTCCAGAAAAC
	Reverse	TACTCTCACGACAGTTGCCAT
HGF	Forward	GCTATCGGGGTAAAGACCTACA
	Reverse	CGTAGCGTACCTCTGGATTGC
